# Green sanctuaries: residential green and garden space and the natural environment mitigate mental disorders risk of diabetic patients

**DOI:** 10.1186/s12916-025-03864-y

**Published:** 2025-01-21

**Authors:** Erxu Xue, Jianhui Zhao, Jingyu Ye, Jingjie Wu, Dandan Chen, Jing Shao, Xue Li, Zhihong Ye

**Affiliations:** 1 Nursing Department, Sir Run Run Shaw Hospital, Zhejiang University School of Medicine, Hangzhou, 310016 China; 2 Department of Big Data in Health Science School of Public Health, The Second Affiliated Hospital and School of Public Health, Zhejiang University School of Medicine, Hangzhou, 310058 China; 3 Department of Epidemiology, School of Public Health, Harbin Medical University, Harbin, 150086 China; 4 Department of Nursing, the Fourth Affiliated Hospital of School of Medicine, and International School of Medicine, International Institutes of Medicine, Zhejiang University, Yiwu, 313098 China; 5 Institute of Nursing Research, School of Medicine, Zhejiang University, Hangzhou, 310058 China

**Keywords:** Diabetes, Green space, Garden space, Natural environment, Mental disorders, Depression disorders, Anxiety disorders

## Abstract

**Background:**

The co-occurrence of diabetes and mental disorders is an exceedingly common comorbidity with poor prognosis. We aim to investigate the impact of green space, garden space, and the natural environment on the risk of mental disorders among the population living with diabetes.

**Methods:**

We performed a longitudinal analysis based on 39,397 participants with diabetes from the UK Biobank. Residential green and garden space modeled from land use data and the natural environment from Land Cover Map were assigned to the residential address for each participant. Cox proportional hazards model was used to analyze the associations between nature exposures and mental disorders of diabetes. Casual mediation analysis was used to quantify indirect effect of air pollution.

**Results:**

During a mean follow-up of 7.55 years, 4513 incident mental disorders cases were identified, including 2952 depressive disorders and 1209 anxiety disorders. Participants with natural environment at 300 m buffer in the second and third tertiles had 7% (HR = 0.93, 95%CI: 0.86–0.99) and 12% (HR = 0.88, 95%CI: 0.82–0.94) lower risks of incident mental disorders compared with those in the first tertile, respectively. The risk of mental disorders incidence among diabetes patients will decrease by 13% when exposed to the third tertile of garden space at 300 m buffer. The natural environment and garden space individually prevented 6.65% and 10.18% of mental disorders incidents among diabetes patients. The risk of incident mental disorders was statistically decreased when exposed to the third tertile of green space at 1000 m buffer (HR = 0.84, 95% CI: 0.78–0.90). Protective effects of three nature exposures against depressive and anxiety disorders in diabetes patients were also observed. Air pollution, particularly nitrogen dioxide, nitrogen oxides, and fine particulate matter, significantly contributed to the associations between nature exposures and mental disorders, mediating 48.3%, 29.2%, and 62.4% of the associations, respectively.

**Conclusions:**

Residential green and garden space and the natural environment could mitigate mental disorders risk in diabetes patients, with air pollution playing a vital mediator. This highlights the potential for local governments to enhance the sustainability of such interventions, grounded in public health and urban planning, through strategic planning initiatives.

**Graphical Abstract:**

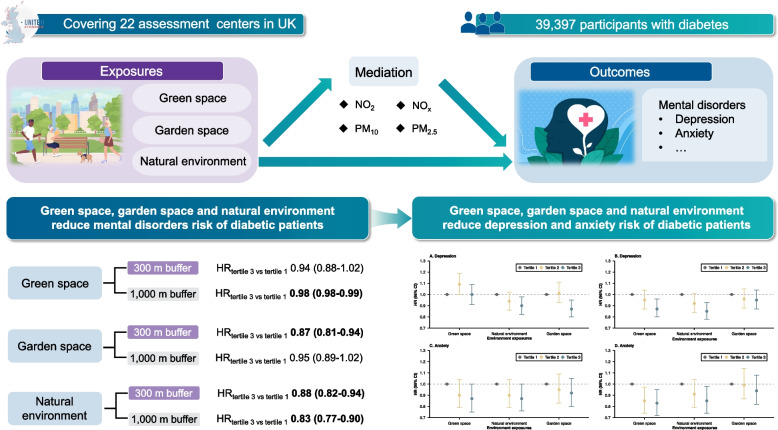

**Supplementary Information:**

The online version contains supplementary material available at 10.1186/s12916-025-03864-y.

## Background

As one of the paramount public health challenges of the twenty-first century, the prevalence of diabetes has incessantly surged over the past few decades, with projections estimating a global diabetic population of 1.31 billion by 2050, corresponding to an annualized rate of change of 3.31% [1]. Diabetes-associated elevated mortality rates result in rising disease burden, thereby imposing a substantial societal and economic burden [2, 3].

The majority of the global burden of diabetes mellitus arises from its complications. As the life expectancy of diabetic patients increases, a spectrum of novel complications is reshaping the landscape of diabetic comorbidities, encompassing cognitive decline, functional impairments, and mental disorders, among others [4, 5]. Previous studies have indicated that individuals with diabetes are at approximately 25% higher risk of developing depression [4, 6], 20% more likely to experience anxiety disorders [7], and face a 38% increased risk of developing mood disorders compared to the global general population [8]. In patients with diabetes, the presence of severe mental illnesses (SMI), included schizophrenia, bipolar disorder, and major depression, is associated with an increased risk of major cardiovascular disease (CVD), as well as approximately a twofold higher risk of cardiovascular-specific mortality and all-cause mortality [9, 10]. Moreover, comorbid mental disorders in diabetic patients are poised to elevate rates of hospitalization and associated costs, as well as frequencies of outpatient emergency visits and overall medical expenditures [11]. On the other hand, mental disorders are linked to worse outcomes in the diabetic population relative to the general population [12]. For example, a study indicated that depression is associated with a 26% increase in all-cause mortality risk within the general population, while this risk escalates to 116% among individuals with diabetes [13]. Moreover, elevated stress or depressive symptoms—either independently or concurrently—significantly elevated the incidence of stroke and CVD mortality among diabetic patients, surpassing that of the general population [14]. Therefore, further identification of relevant risk factors to prevent the onset of mental disorders in individuals living with diabetes remains an essential endeavor.

Recently, there has been a growing emphasis on investigating the influence of the physical environment—the geographic area and surrounding factors—on the incidence of mental disorders. Growing evidence supports the critical impact of biodiversity on human health and well-being, with one key mechanism being the association between exposure to environmental typologies, such as green space and mental health [15–18]. Green space, garden space, and the natural environment typically refer to open space for greening or leisure, domestic gardens, and residential non-building space, respectively [19, 20]. There is accumulating evidence from cross-sectional and prospective studies that exposure to green and garden space and the natural environment has beneficial effects on mental health [20], especially for depressive and anxiety disorders [21]. Under the biodiversity-health framework, these beneficial health outcomes may be achieved through three pathways: (i) harm reduction (e.g., reduction of exposure to air and noise pollution); (ii) restoring capacities (e.g., attention restoration, stress recovery); and (iii) capacity building (e.g., promotion of physical activity) [22, 23]. However, given the elevated prevalence of mental disorders among diabetic patients, there is currently insufficient evidence to substantiate a causal relationship between exposure to residential green and garden space and the natural environment with incident mental events in individuals with diabetes.

Herein, based on the prevailing global burden of mental disorders [24, 25], we initially explored the impact of residential green and garden space and the natural environment on the incidence of selected mental disorders among individuals with diabetes in a prospective cohort. Considering the higher prevalence rate in the population with diabetes [26, 27], this study also examined certain specific mental disorders, including depressive disorders and anxiety disorders. Furthermore, we focused on the potential effect modification and mediation role of air pollutants in the associations.

## Methods

### Study design and participants

The study used data from the UK Biobank (UKB, https://www.ukbiobank.ac.uk/), a large prospective cohort study with more than 500,000 people aged 37–73 years from 22 assessment centers across England, Wales, and Scotland, which has been documented in detail elsewhere [28]. The baseline survey was conducted during 2006–2010, which collected extensive data on health-related information of the participants through questionnaires, physical measures, sampling assays, genotyping, and longitudinal follow-up of various health outcomes.

For the primary analysis investigating associations of green and garden space and the natural environment with incident mental disorders, we initially selected patients who had received a diagnosis of diabetes, irrespective of the type of diabetes. Specifically, individuals with diabetes were identified via hospital inpatient data recorded in the International Classification of Diseases 10th revision (ICD-10). We included 47,823 individuals with diabetes and excluded participants with missing nature exposure data (*n* = 4623) and participants diagnosed with mental disorders within the baseline period (*n* = 3803). Finally, 39,397 individuals with diabetes were included in the primary analysis (Additional file 1: Fig. S1).

### Assessment of exposure

Consistent with previous research [20, 29], we used land use data from the 2005 Generalized Land Use Database (GLUD) for England provided by the Department for Communities and Local Government of the Government of the UK (https://www.gov.uk/government/statistics) to estimate residential green and garden space. GLUD classification of high-resolution land parcels was distributed to 32,482 lower-layer super output areas (LSOAs) across England, each encompassing approximately 1500 residents (mean area c.4 km^2^). The GLUD divides the total land cover in each LSOA into nine categories: green space, domestic gardens, fresh water, domestic buildings, nondomestic buildings, roads, paths, railways, and others (largely hard standing). The percentages of residential green and garden space, which were classed as “Green space” and “Garden space,” were proportions of the total percentage of all land-use types and with home location data buffered at 300 m and 1000 m [29, 30].

The data on the distribution of the natural environment (home location buffer classed as “Natural environment”) were collected from the Land Cover Map (LCM) 2007 of the Centre for Ecology and Hydrology (CEH) [30, 31]. The LCM 2007 product included 23 land cover classes (broadleaved woodland, coniferous woodland, arable and horticulture, improved grassland, rough grassland, neutral grassland, calcareous grassland, acid grassland, fen marsh and swamp, heather, heather grassland, bog, montane habitats, inland rock, saltwater, freshwater, supra-littoral rock, supra-littoral sediment, littoral rock, littoral sediment, saltmarsh, suburban, urban) with Class 1–21 reclassified as the natural environment. Notably, Class 22–23 encompass buildings and gardens, which differs from the definition of GLUD.

### Ascertainment of outcome

The main outcomes of this study were the incidence of mental disorders, depressive disorders, and anxiety disorders. Individuals diagnosed with specific mental disorders at baseline and subsequent follow-up were confirmed based on ICD-10. The UK’s National Health Service (NHS) gathers regular data on admissions, including information about all patients admitted to NHS hospitals. The hospital records were connected to UKB members by anonymized numeric participant identification numbers. The diagnosis of mental disorders will be given by psychiatrists. Based on the current global burden of mental disorders [24, 25], we selected 14 specific mental illnesses, including depressive disorders, anxiety disorders, schizophrenia, schizotypal disorder, persistent delusional disorders, acute polymorphic psychotic disorder without symptoms of schizophrenia, induced delusional disorder, schizoaffective disorders, other nonorganic psychotic disorders, unspecified nonorganic psychosis, manic episode, bipolar affective disorder, and post-traumatic stress disorder cases. The identification of mental disorders was based on the ICD-10 codes, and the detailed diagnostic code for mental disorders can be found in Additional file 1: Table S1. Considering the higher prevalence rate in the population with diabetes [26, 27], this study also examined certain specific mental disorders, including depressive disorders and anxiety disorders. The time-to-event for participants was calculated from the date of recruitment to the date of the first diagnosis of mental disorder or date of death, date of loss to follow-up, or end of follow-up, whichever came first.

### Assessment of covariates

Possible confounding factors in this study were selected by reviewing previous studies related to mental disorders [21, 32], including age, sex, ethnicity, household income, educational levels, employment status, smoking status, alcohol intake, physical activity, healthy diet pattern, and healthy sleep pattern. Physical activity was measured by the metabolic equivalent task (MET). More details of the covariate process are presented in Additional file 1: Table S2 [33, 34]. Furthermore, four air pollutants, nitrogen oxides (NO_x_), nitrogen dioxide (NO_2_), particulate matter (PM) with aerodynamic diameter ≤ 10 µm (PM_10_), and PM with aerodynamic diameter ≤ 2.5 µm (PM_2.5_), were selected according to the air pollution criteria by the World Health Organization [35] and European Commission [36] based on the data availability of the UKB. Air pollution estimates for PM_2.5_ and NO_x_ were available for the year 2010 in UKB, whereas NO_2_ (2005–2007 and 2010) and PM_10_ (2007 and 2010) had the exposure data for several years; data from 2010 were used uniformly in our analysis.

### Statistical analyses

Baseline characteristics of individuals with diabetes were summarized as means with standard deviations (SD) for continuous variables and percentages for categorical variables. Continuous variables were assessed for statistical differences using Mann–Whitney *U* tests. Categorical variables were evaluated for differences using the ${\chi }^{2}$ test. Correlation between green space, garden space, and natural environment and air pollutants was reported using Pearson correlation coefficients. For variables with a missing rate of > 5% (i.e., household income), missing data were coded as an independent category; otherwise, missing data were imputed as median values for continuous variables or mode values for categorical variables. Three environment variables were treated as continuous and categorical variables. Tertiles of exposures were used as cutoffs, with the first tertile (the lowest) set as the reference group [20]. We also calculated hazard ratios (HRs) by each 5% increment in the exposures.

We used Cox proportional hazard models to estimate HRs and 95% confidence intervals (CIs) for the associations of green space, garden space, and the natural environment at 300 and 1000 m buffer with incident mental disorders among the population living with diabetes. We excluded events of other mental disorders when separately analyzing the relationship between nature exposures and either incident depressive disorders or incident anxiety disorders. Models were incrementally adjusted: model 1 adjusted for age, sex, and ethnicity, while model 2 additionally adjusted for household income, education, employed, smoking, alcohol consumption, physical activity (MET), healthy diet pattern, and healthy sleep pattern. Linear trends were tested using each categorical variable (three-categorical) as continuous variables. The proportional hazards assumption was tested with Schoenfeld residuals (Additional file 1: Figs. S2-S4). The population attributable fraction (PAF) was calculated to reflect the proportion of the events that could be avoided by eliminating the lowest tertile level of nature exposures. To delineate the linear and non-linear associations between nature exposures and incident mental disorders, we further constructed restricted cubic spline (RCS) models with Harrell’s knots placed at the 5th, 35th, 65th, and 95th percentiles of green, garden space, and natural environment while adjusting for all confounders. For significant associations observed in the primary analysis, we further conducted mediation analysis as a secondary analysis to explore whether ambient air pollutants mediated the associations between nature exposures and mental disorders incidence risk. Mediation analysis distinguishes the direct effect of specific nature exposures on the risk of mental disorders and the indirect effect mediated by air pollutants. The point estimates and 95% CIs of the proportion of exposure effect attributable to the mediators were calculated using the difference method by comparing the exposure effect estimation from the full model (i.e., indirect effect/(indirect + direct effect)) [37, 38]. Mediation analysis was conducted with 1000 bootstrap samples to estimate bias-corrected bootstrap CI [39].

To explore the potential heterogeneity of the association between green and garden space and the natural environment with mental health outcomes, stratified analyses by age (< 65 years vs. ≥ 65 years), sex (female vs. male), ethnicity (white vs. non-white), household income (low vs high), education level (college vs. non-college), employment status (currently unemployed vs. currently employed), smoking status (never vs. previous/current), alcohol drinking (none to moderate vs. heavy), physical activity (high vs. low), healthy diet (unhealthy or healthy), and healthy sleep pattern (unhealthy or healthy) were performed. The P for interaction was calculated by testing the change of goodness-of-fit before and after allowing a multiplication term of the green space, garden space, and natural environment (three-categorical) and these covariates. In sensitivity analyses, we assessed the robustness of our findings using different model specifications. First, given that type 2 diabetes (T2D) accounts for over 95% of all diabetes cases globally [40], we re-conducted the primary analysis to investigate the association between nature exposures and mental disorders in patients with T2D. Second, to assess whether the protective effects of the nature exposures are significantly greater for the diabetic sample, the primary analysis was repeated on the sample without diabetes. Third, based on the fully adjusted model, we further restricted to individuals who had lived at the current address more than 10 years before baseline. Fourth, to mitigate the potential impact of reverse causality, we re-examined the associations between nature exposures and mental disorders incidence after excluding participants with mental disorders events within 1 year of follow-up; fifth, we re-ran our primary analyses after excluding the participants with missing data on covariates to examine the associations between exposure to green and garden space and the natural environment and mental disorders. Sixth, the crude hazard ratios were calculated as a reference point to assess the magnitude of variation in results across models. Lastly, as previous studies have suggested that ambient air pollution is also associated with mental illness among diabetes patients [41], we validated results in new models with further adjustment for NO_2_, NO_x_, PM_10_, and PM_2.5_. These air pollutants would be added individually to the model in turn and finally together to assess the robustness of the results.

Two-sided *P* values < 0.05 were considered to be statistically significant. All statistical analyses were conducted using R software (version 4.3.3).

## Results

### Baseline characteristics

The characteristics of the study population are presented in Table 1. The mean ± SD age of the 39,397 recruited participants (female: 40.1%, male: 59.9%) was 59.62 (7.19) years. During an average follow-up period of 7.55 years, we documented 4513 incident cases of mental disorders, including 2952 cases of depressive disorders, and 1209 cases of anxiety disorders (Additional file 1: Table S3). The proportion of individuals lost to follow-up was 0.08% (30 cases). Participants who developed mental disorders were slightly younger at baseline, more likely to be female, had lower socioeconomic status (e.g., lower educational level and higher rate of unpaid employment), and had unhealthy lifestyles (e.g., low physical activity, unhealthy diet and sleeping patterns). The average levels of residential green space and natural environment at the 300 m buffer were lower than the exposure levels at the 1000 m buffer, while the mean level of garden space was higher at the 300 m buffer (Additional file 1: Fig. S5 and details in Table S4).

**Table 1:** Baseline characteristics of the study participants in total and by incident mental disorders

Characteristics	All cohort (*N* = 39,397)	Non-cases (*N* = 34,884)	Incident mental disorders cases (*N* = 4,513)	*P* value
Age, years	59.62 (7.19)	59.73 (7.13)	58.76 (7.53)	< 0.001
Sex				
Female, *n* (%)	15,803 (40.1)	13,531 (38.8)	2272 (50.3)	< 0.001
Male, *n* (%)	23,594 (59.9)	21,353 (61.2)	2241 (49.7)	
Ethnicity				
White, *n* (%)	34,844 (88.4)	30,823 (88.4)	4021 (89.1)	0.151
Non-white, *n* (%)	4553 (11.6)	4061 (11.6)	492 (10.9)	
Education				
College/university degree, *n* (%)	8517 (21.6)	7659 (22.0)	858 (19.0)	< 0.001
Other, *n* (%)	30,880 (78.4)	27,225 (78.0)	3655 (81.0)	
Household income				
Low, *n* (%)	27,215 (69.1)	23,983 (68.8)	3232 (71.6)	< 0.001
High, *n* (%)	4577 (11.6)	4259 (12.2)	318 (7.0)	
Unknown, *n* (%)	7605 (19.3)	6642 (19.0)	963 (21.3)	
Employment				
Currently employed, *n* (%)	16,711 (42.4)	15,259 (43.7)	1452 (32.2)	< 0.001
Currently unemployed, *n* (%)	22,686 (57.6)	19,625 (56.3)	3061 (67.8)	
Cigarette smoking				
Never, *n* (%)	18,271 (46.4)	16,350 (46.9)	1921 (42.6)	< 0.001
Former, *n* (%)	16,367 (41.5)	14,511 (41.6)	1856 (41.1)	
Current, *n* (%)	4759 (12.1)	4023 (11.5)	736 (16.3)	
Alcohol consumption				
None to moderate, *n* (%)	31,685 (80.4)	27,934 (80.1)	3751 (83.1)	< 0.001
Heavy, *n* (%)	7712 (19.6)	6950 (19.9)	762 (16.9)	
Physical activity (MET) ^a^				
Low, *n* (%)	15,453 (39.2)	13,612 (39.0)	1841 (40.8)	< 0.001
High, n (%)	12,515 (31.8)	11,294 (32.4)	1221 (27.1)	
Unknown, *n* (%)	11,429 (29.0)	9978 (28.6)	1451 (32.2)	
Healthy diet				
Unhealthy, *n* (%)	25,764 (65.4)	22,886 (65.6)	2878 (63.8)	< 0.001
Healthy, *n* (%)	10,714 (27.2)	9480 (27.2)	1234 (27.3)	
Unknown, *n* (%)	2919 (7.4)	2518 (7.2)	401 (8.9)	
Healthy sleep				
Unhealthy, *n* (%)	16,287 (41.3)	14,096 (40.4)	2191 (48.5)	< 0.001
Healthy, *n* (%)	14,761 (37.5)	13,497 (38.7)	1264 (28.0)	
Unknown, *n* (%)	8349 (21.2)	7291 (20.9)	1058 (23.4)	

Data are presented as mean (SD) and number (%) for continuous and categorical variables, respectively

*Abbreviations: MET* metabolic equivalent of task, *SD* standard deviation

^a^Physical activity: MET scores (range 0–21, positively correlated with weekly physical activity) below and above the cohort median represent low and high physical activity, respectively

### The associations of green and garden space and the natural environment with the risk of mental disorders among individuals with diabetes

When investigating the associations of the natural environment with risk of mental disorders among individuals with diabetes, the risk of mental disorders would be 7% lower (HR 0.93, 95%CI: 0.86–0.99) at the second tertile at the 300 m buffer and 12% lower at the highest tertile (HR 0.88, 95%CI: 0.82–0.94), and the trend test was also statistically significant (Table 2). At 1000 m buffer distances, relationships between natural environment and mental health outcomes showed a similar trend (tertile 2: HR 0.91, 95%CI: 0.84–0.97; tertile 3: HR 0.83, 95%CI: 0.77–0.90) (Table 3). There were significant negative associations between the exposure to garden space and mental disorders risk of diabetic patients (Table 2). And the HR at third tertile of garden space at 300 m buffer was 0.87 (95%CI: 0.81–0.94). Participants with diabetes exposed to higher green space level had a lower risk of mental disorders compared to the lowest tertiles of green space at 1000 m buffer (HR 0.90, 95%CI: 0.84–0.97 (tertile 2); HR 0.84, 95%CI: 0.78–0.90 (tertile 3); *P*_trend_ < 0.001) (Table 3).

**Table 2:** Associations of exposure to green, garden space, and natural environment at 300 m buffer with incident mental disorders among people with diabetes

	***N***	**Cases/person-years**	**Model 1** **HR (95% CI)**	***P* value**	**Model 2** **HR (95% CI)**	***P* value**	**PAF (%)** **(95% CI [%])**
**Green space**							5.20 (3.25–7.16)
Tertile 1	13,133	1521/98,595	Reference		Reference		
Tertile 2	13,132	1566/99,156	1.02 (0.95–1.10)	0.580	1.01 (0.94–1.08)	0.889	
Tertile 3	13,132	1426/99,592	0.93 (0.87–1.00)	0.053	0.94 (0.88–1.02)	0.122	
* P* for trend	-	-	-	0.055	-	0.124	
Per 5% increment	-	-	0.99 (0.98–0.99)	< 0.001	0.99 (0.98–1.00)	0.002	
**Natural environment**							6.65 (4.70–8.61)
Tertile 1	13,263	1632/99,356	Reference		Reference		
Tertile 2	13,051	1482/98,257	0.92 (0.86–0.99)	< 0.001	0.93 (0.86–0.99)	0.031	
Tertile 3	13,083	1399/99,729	0.85 (0.79–0.92)	< 0.001	0.88 (0.82–0.94)	< 0.001	
* P* for trend	-	-	-	< 0.001	-	< 0.001	
Per 5% increment	-	-	0.99 (0.98–0.99)	< 0.001	0.99 (0.98–1.00)	0.004	
**Garden space**							10.18 (8.25–12.12)
Tertile 1	13,133	1600/98,540	Reference		Reference		
Tertile 2	13,133	1562/99,899	0.96 (0.90–1.03)	< 0.001	0.98 (0.91–1.05)	0.502	
Tertile 3	13,131	1351/98,904	0.84 (0.78–0.90)	< 0.001	0.87 (0.81–0.94)	< 0.001	
* P* for trend	-	-	-	< 0.001	-	< 0.001	
Per 5% increment	-	-	0.98 (0.97–0.99)	< 0.001	0.98 (0.97–0.99)	0.001	

Model 1: adjusted for age, sex, and ethnicity. Model 2: adjusted for age, sex, ethnicity, household income, education, employed, smoking, alcohol consumption, physical activity (MET), healthy diet pattern, and healthy sleep pattern

*Abbreviations: CI* confidence interval, *HR* hazard ratio, *MET* metabolic equivalent of task, *PAF* population attributed fraction

**Table 3:** Associations of exposure to green, garden space, and natural environment at 1000 m buffer with incident mental disorders among people with diabetes

	***N***	**Cases/person-years**	**Model 1** **HR (95% CI)**	***P* value**	**Model 2** **HR (95% CI)**	***P* value**	**PAF (%)** **(95% CI (%)**
**Green space**							9.53 (7.59–11.46)
Tertile 1	13,133	1649/98,669	Reference		Reference		
Tertile 2	13,132	1503/99,157	0.90 (0.84–0.97)	0.005	0.90 (0.84–0.97)	0.005	
Tertile 3	13,132	1361/99,516	0.81 (0.76–0.88)	< 0.001	0.84 (0.78–0.90)	< 0.001	
P for trend	-	-	-	< 0.001	-	< 0.001	
Per 5% increment	-	-	0.98 (0.97–0.99)	< 0.001	0.98 (0.98–0.99)	< 0.001	
**Natural environment**							10.58 (8.65–12.51)
Tertile 1	13,138	1662/98,714	Reference		Reference		
Tertile 2	13,128	1506/99,154	0.90 (0.84–0.96)	< 0.001	0.91 (0.84–0.97)	0.006	
Tertile 3	13,131	1345/99,474	0.80 (0.74–0.86)	< 0.001	0.83 (0.77–0.90)	< 0.001	
P for trend	-	-	-	< 0.001	-	< 0.001	
Per 5% increment	-	-	0.98 (0.98–0.99)	< 0.001	0.98 (0.98–0.99)	< 0.001	
**Garden space**							3.27 (1.30–5.24)
Tertile 1	13,134	1567/99,151	Reference		Reference		
Tertile 2	13,132	1491/99,062	0.95 (0.89–1.03)	< 0.001	0.96 (0.89–1.03)	0.252	
Tertile 3	13,131	1455/99,130	0.93 (0.87–1.00)	< 0.001	0.95 (0.89–1.02)	0.185	
P for trend	-	-	-	0.046	-	0.182	
Per 5% increment	-	-	0.99 (0.97–1.00)	0.045	0.99 (0.98–1.00)	0.154	

Model 1: adjusted for age, sex, and ethnicity. Model 2: adjusted for age, sex, ethnicity, household income, education, employed, smoking, alcohol consumption, physical activity (MET), healthy diet pattern, and healthy sleep pattern

*Abbreviations: CI* confidence interval, *HR* hazard ratio, *MET* metabolic equivalent of task, *PAF* population attributed fraction

**Fig. 1:**
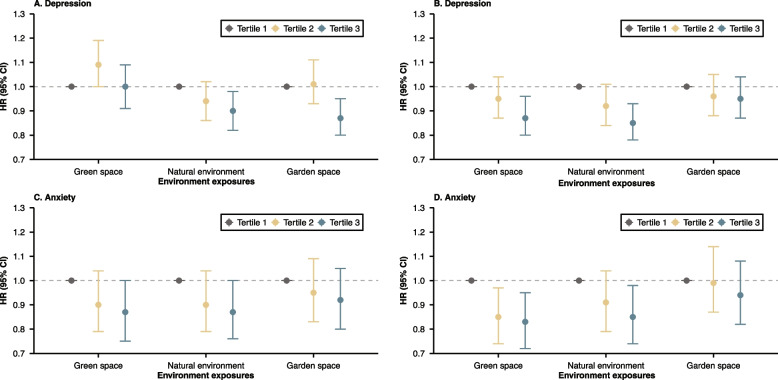
Associations of exposure to green and garden space and natural environment at 300 and 1000 m buffer with incident depression (**A** and **B**) and anxiety disorders (**C** and **D**) among people with diabetes

**Fig. 2:**
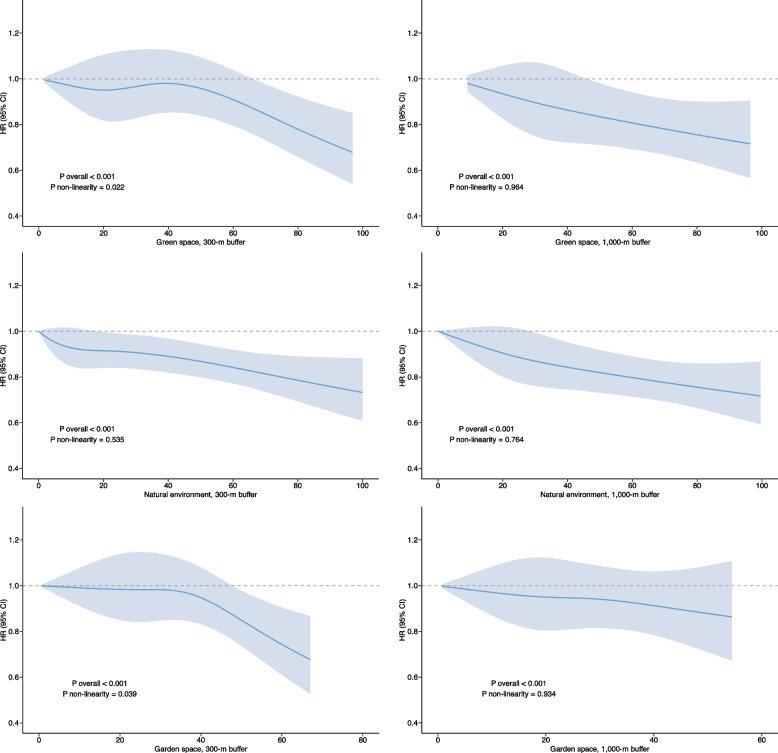
Exposure–response curves between green and garden space and natural environment with incident mental disorders

Figure 1 illustrates the relationship between green, garden space and natural environment with the risk of depressive and anxiety disorders. The detailed results were presented in Additional file 1: Tables S5-S6. Compared with the lowest tertile of the natural environment and garden space at the 300 m buffer, the risks of depressive disorders were significantly lower in the highest tertile (10% (HR 0.90, 95%CI: 0.82–0.98) lower at natural environment, 13% (HR 0.87, 95%CI: 0.80–0.95) lower at garden space, both *P* trend < 0.05). For anxiety disorders, 13% and 17% lower for third tertile at green space and 13% and 15% lower for third tertile at the natural environment, with similar results seen for both the 300 m and 1000 m buffer. Figure 2 depicted the exposure–response relationships between green, garden space, and natural environment at 300 m and 1000 m buffer and incident mental disorders. The relationships showed a protective effect at higher nature exposures, and no saturation effect was observed. Except for the non-linear relationship observed between green and garden space at 300 m buffer and the incidence of mental disorders of the diabetic population (*P* non-linearity = 0.022, 0.039, respectively), all other factors exhibit a linear correlation (all *P* non-linearity > 0.05).

### Mediation analysis

According to the results of correlation analysis (Additional file 1: Table S7), we elected four air pollutants as potential mediators concerning the association between green space and natural environment exposure with incident mental disorders of diabetic patients. The mediation analyses suggested that air pollution played a major mediator role in the associations of green space and natural environment within both studied buffers with incident mental disorders (Additional file 1: Table S8). Specifically, the association between green space at 300 m buffer and mental disorders was 77.1%, 44.3%, and 92.1%, mediated by the decreased NO_2_, NO_x_, and PM_2.5_. The association between natural environment at 300 m buffer and mental disorder was 48.3%, 29.2%, and 62.4%, mediated by the decreased NO_2_, NO_x_, and PM_2.5_, respectively. We did not observe significant mediation effects of PM_10_, and this holds true for both green space and natural environment. Moreover, we also observed that 45.8% of the associations between green space at 1000 m buffer and mental disorders among individuals with diabetes were mediated by PM_2.5_, whereas 35.8% of the associations between the natural environment at 1000 m buffer and mental disorders among individuals with mental disorders were mediated. NO_2_, NO_x_, and PM_10_ were not significant mediators between green space, natural environment at 1000 m buffer, and mental disorders risk (all *P* indirect effect > 0.05).

### Subgroups and sensitivity analyses

Subgroup analyses showed that the protective effect of green space and natural environment at 300 m buffer on mental disorders tended to be stronger in the individuals with heavy alcohol consumption (*P* for interaction < 0.05), but such pattern was not observed for garden space (Additional file 1: Tables S9-11). Stronger associations between the natural environment at 300 m buffer existed for the former or current smokers. No consistent evidence of potential modifying effects was observed for other factors.

In sensitivity analysis, among patients with T2D, green space at 1000 m buffer, garden space at 300 m buffer, and natural environment at both 300 m and 1000 m buffer remained positively and significantly associated with a reduced risk of mental disorders (Additional file 1: Table S12). Compared with those without diabetes, the effect of nature exposures on the reduction of mental disorders is overall more pronounced in the diabetic population (Additional file 1: Table S13). The results were consistent with primary findings or maintained similar magnitude when restricted to individuals who had at least 10 years of residence at baseline (Additional file 1: Table S14), excluding participants with mental disorders events within 1 year of follow-up (Table S15), and excluding the participants with missing data on covariates (Additional file 1: Table S16). The crude HRs and 95% CIs of incident mental disorders associated with green space, garden space, and natural environment at 300 m or 1000 m buffer among people with diabetes was shown in Additional file 1: Table S17. In analyses with additional adjustment for four ambient air pollutants including NO_2_, NO_x_, PM_10,_ and PM_2.5_ (individually or collectively), the main results remained robust (Additional file 1: Tables S18 and S19).

## Discussion

This study first indicated that exposure to green and garden space and the natural environment confers a protective effect against incident mental disorders among individuals with diabetes, preventing 5.20%, 6.65%, and 10.18% of mental disorder events, respectively. Exposure to the third tertile of the natural environment at 300 m and 1000 m buffer can respectively reduce the risk of depressive disorders by 10% and 15% among diabetics. The risk of anxiety disorders among diabetic patients was reduced by 13% and 17% when exposed to the third tertile of green space at 300 m and 1000 m buffer, respectively. Additionally, we found that NO_2_, NO_x_, and PM_2.5_ mediated 29.2% to 92.1% of the associations between green space and natural environment with the risk of mental disorders incidence among participants living with diabetes.

Our study demonstrated that long-term exposure to green space significantly protects against mental disorders, including both depressive and anxiety disorders, in diabetic patients. First, a higher rate of green space will enhance physical and social activities among diabetic patients [42], thereby increasing time spent with family, friends, and neighbors [43], which contributes to a more optimistic mood and positively influences the mental health of diabetic individuals. Additionally, improvement of air quality [44], and reduction of noise pollution [45], also represent intrinsic mechanisms through which green space can prevent mental disorders in diabetic patients. Research indicated that vegetation can reduce gaseous air pollutants through deposition to plant cuticles and stomatal uptake [46, 47]. Vegetations directly influence particulate matter in the atmosphere by intercepting particles, emitting substances (e.g., pollen), and resuspending captured on the plant surface [48, 49]. While certain particles may be absorbed by vegetation, the majority of intercepted particulates are retained on the plant surface [48]. Previous studies have indicated a potential causal relationship between long-term exposure to air pollution and the incidence of mental disorders in diabetic patients [41]. Therefore, the enhancement of air quality through green space would significantly benefit this vulnerable population. Moreover, the results of the subgroup analysis indicated that the protective effect of green space in reducing the risk of mental disorders of diabetes was more pronounced in those with heavy alcohol consumption. We know that alcohol can induce neurochemical alterations in the central nervous system and modify the gut microbiome, consequently contributing to the development of mental disorders [50]. Thus, nature-based intervention practices could yield effects greater than initially anticipated, particularly within diabetic population with heavy alcohol consumption.

We also found that long-term garden space exposure significantly reduced the risk of mental disorders among individuals with diabetes, including depressive disorders. The initial objective assessment of residential garden access and its impact on health outcomes indicated a positive correlation between access to and utilization of garden space and self-reported health [51]. Recently, Roscoe et al. have also reported that private residential garden cover has a stronger effect on mortality than total green space cover [52]. In our study, the impact of garden space at 300 m buffer on the reduction of mental disorders risk among diabetes patients was greater than that of green space, with a similar trend observed at 1000 m buffer, although it was not statistically significant. This may be related to the accessibility of garden space, more frequent exposure to these areas, and prolonged duration spent within them [53]. Further in-depth research is warranted into the mechanisms underlying the greater health benefits brought about by such garden space. As suggested by de Bell and colleagues, the act of viewing, accessing, and/or using residential gardens may yield specific health benefits, such as through psychological and/or physical activity mechanisms, for instance, gardening [51].

Furthermore, our findings align with the latest prospective longitudinal study to date, indicating a protective effect of the natural environment at either 300 m or 1000 m against mental disorders, depressive, or anxiety disorders among the diabetic population [20]. Similar to findings reported in a meta-analysis integrating 33 studies, short-term exposure to the natural environment exhibits a marginal effect in reducing depressive symptoms [54]. The quantitative estimation by Zhang et al. suggested that natural exposure may effectively mitigate human anxiety [55]. During the COVID-19 pandemic, nature contact and appreciation of nature significantly reduced the risk of anxiety and depression [56]. Notably, the natural environment may reduce the risk of mental disorders in individuals with diabetes by catalyzing nature experience [57]. As a determinant of mental health, nature experience has been shown to enhance happiness and subjective well-being, foster positive social interactions, strengthen a sense of meaning and purpose in life, improve disease self-management abilities, and alleviate psychological distress such as negative emotions [58]. Our findings indicated that in the diabetic population who are heavy drinkers and former and current smokers, the natural environment more effectively mitigates the risk of mental disorders incidence. This further provides direction for implementing nature-based interventions in populations with diabetes who are more susceptible to mental illness.

The biodiversity-health framework may aid in elucidating the potential mechanisms underlying the impact of three distinct exposure measures across two different buffers on mental health outcomes [59]. Domestic garden as a small ecosystem enhance human mental health at a small scale, including facilitating recovery from stress, increasing green space perception and satisfaction with the living environment, and protecting against environmental factors such as extreme heat [60]. The time spent in garden space and the frequency of visits significantly influence mental health [61]; thus, it can be understood that the garden space at 300 m buffer is more effective in reducing the risk of mental diabetes among diabetic patients. Previous studies have shown that biodiversity may lead to better mental health outcomes [17], such as where bird diversity and habitat diversity are greater [62], which could be realized within green space and natural environment. Moreover, the natural environment includes blue space, characterized primarily by water and accessible to humans, which may further enhance biodiversity [63]. This could be a significant underlying mechanism by which the natural environment of the two buffer zones in our study are able to mitigate the risk of mental disorders, particularly depression. Furthermore, the exposure–response curves showed that the effects of green space, garden space, and natural environment on mental disorders in diabetes patients tend to be more pronounced at greater exposure, which suggested the need for continued greenery and urban planning to protect the mental health of the population living with diabetes. We also found that air pollution (NO_2_, NO_x_, PM_2.5_) played a crucial mediating role in the relationship between nature exposures and mental disorders among diabetes patients. Prior studies have clearly indicated that the presence of green space and favorable natural surroundings can substantially enhance air quality and confer mental well-being advantages [64]. Meanwhile, it has been found that exposure to air pollutants can increase the risk of mental disorders such as depressive and anxiety disorders [65]. Therefore, combining our findings with previous research, we believe that the protective effect of long-term exposure to green space, garden space, and natural environment on the risk of mental disorders among individuals with diabetes is, to some extent, accomplished through the reduction of air pollutants. This underscores the importance of adhering to public health policies aimed at reducing air pollution concentrations. However, it is also worth acknowledging a common counterargument, which is that the impact of green space on air pollutant concentrations is highly context-specific, with some experimental and modeling studies reporting relatively low effects [66]. Finally, relational and collective restoration [43], social support [67], relative deprivation, and physical activities [68, 69], as well as noise, aesthetics, and satisfaction with recreational opportunities [70], have all been shown to mediate the relationship between residential green space, natural environment, and mental health. Therefore, the formulation of comprehensive urban green space planning policies that holistically consider various mediating factors will contribute to maximizing the health benefits of urban green space.

Interestingly, our findings suggested that the protective effects of green space, garden space, and natural environment on mental health are more pronounced in individuals with diabetes compared to general population or those without diabetes. Additionally, in the study by Liu et al., green space within two buffer distances did not significantly reduce the risk of mental disorders in middle-aged and elderly individuals [20]. However, our findings demonstrated that green space at 1000 m buffer effectively mitigates the risk of mental disorders in patients with diabetes. Similarly, the protective effect of the natural environment within the two buffers on mental health is more pronounced in diabetic patients compared to the general population [20]. Recent research indicated that garden space does not offer significant protective effects against late-onset schizophrenia in the general population [71]. However, our findings reveal a significant association between garden space at 300 m buffer and a reduced risk of mental disorders, particularly depression disorders, among the population living with diabetes. Thus, nature exposures should be considered a critical factor in preventing the development of mental disorders in diabetes patients. Integrating this consideration into diabetes management would maximize public health benefits. The integration of specific activities in nature with the science of medicine in the care and management of diabetes could represent a promising tool for prevention as the field of nature-based medicine continues to grow [72]. Nature-based lifestyle interventions, such as community gardens (also known as allotment gardens) and park prescriptions [73–76], can enhance opportunities for outdoor physical activity, foster social networks of neighbors with a shared interest, and promote an activity that promotes cognitive stimulation and fosters meaningful experiences [77], collectively reducing the risk of mental disorders in individuals with diabetes. Thus, integrating nature-based green prescriptions into the clinical practice of primary care providers may not only reduce the risk of mental disorders in patients with diabetes but also improve key behavioral risk factors for other chronic complications [78].

### Limitations

We should acknowledge certain limitations. First, as with the majority of large-scale epidemiological studies, participants in the UKB were recruited on a voluntary basis, thereby rendering selection bias inevitable. Second, exposure to green space, garden space, and natural environment, as well as air pollution was assessed only at baseline, which may have led to misclassification if an individual changed residential location. While residential environments in developed countries like the UK generally do not undergo significant changes [79], the possibility of alterations in residential environments still exists. In addition, the estimated air pollution concentrations at baseline may not fully capture long-term air pollution exposure over an extended follow-up period; previous studies have indicated the stability of air pollution exposure during prolonged follow-up. Therefore, this exposure estimate is considered consistently reliable [80–83]*.* Third, despite adjusting for potential confounding factors as comprehensively as possible in this study, residual confounding bias cannot be completely ruled out. Additionally, certain covariates may vary over the course of follow-up, potentially leading to misclassification bias. Fourth, we only gathered data on green space, garden space, and natural environment within 300 and 1000 m buffers; more detailed data within other buffer distances were not available. This emphasizes the requirement for employing advanced techniques to achieve accurate exposure assessment for each individual. Fifth, the persistently high prevalence of other psychiatric disorders, including psychotic and bipolar disorders, is indeed concerning. However, only 12 cases of psychotic disorders and 98 cases of bipolar disorders were identified, thereby limiting our ability to longitudinally examine the association between nature exposures and the risk of other specific mental disorders among participants living with diabetes. Sixth, the baseline survey conducted over four years may lead to potential cohort effects and introduce biases related to the specific timing of entry into the survey, thereby resulting in bias in longitudinal comparisons. Finally, the large number of statistical analyses conducted in this study increases the risk of type I error; thus, the relationship between nature exposures and mental disorders should be interpreted with caution.

## Conclusions

Long-term exposure to residential green, garden space, and the natural environment was associated with a decreased risk of incident mental disorders, as well as incident depressive and anxiety disorders among individuals with diabetes, with air pollutants such as NO_2_, NO_x_, and PM_2.5_ serving as important mediating factors. This highlights the potential for local governments to enhance the sustainability of such interventions, grounded in public health and urban planning, through strategic planning initiatives. This approach can lead to broader improvements in mental health among vulnerable populations like those with diabetes.

## Supplementary Information

Additional file 1: Fig. S1. Flow chart of the inclusion of the participants. Fig. S2. The verification of the proportional hazard assumption in fully adjusted model examined the relationship between green space and incident mental disorders. Fig. S3. The verification of the proportional hazard assumption in fully adjusted model examined the relationship between garden space and incident mental disorders. Fig. S4. The verification of the proportional hazard assumption in fully adjusted model examined the relationship between natural environment and incident mental disorders. Fig. S5. Distribution of residential green space, garden space, and natural environment at baseline. Table S1. ICD-10 codes assigned for mental disorders. Table S2. Definitions for covariates. Table S3. Mental disorders events from 39,397 participants with diabetes in the UK Biobank. Table S4. Distribution of residential green space, garden space, and natural environment at baseline. Table S5. Associations between nature exposures at 300 or 1000 m buffer and incidence of depressive disorders among people with diabetes. Table S6. Associations between nature exposures at 300 or 1000 m buffer and incidence of anxiety disorders among people with diabetes. Table S7. Correlations between nature exposures (Spearman correlation coefficients). Table S8. Mediation analysis in associations of nature exposures at 300 m and 1,000 m buffer with incidence of mental disorders among participants with diabetes. Table S9. Associations between exposure to green space at 300 m buffer and incidence of mental disorders among people with diabetes in stratified analyses. Table S10. Associations between exposure to natural environment at 300 m buffer and incidence of mental disorders among people with diabetes in stratified analyses. Table S11. Associations between exposure to garden space at 300 m buffer and incidence of mental disorders among people with diabetes in stratified analyses. Table S12. Associations between nature exposures at 300 or 1000 m buffer and incidence of mental disorders among people with type 2 diabetes. Table S13. Associations between nature exposures at 300 or 1000 m buffer and incidence of mental disorders among people without diabetes. Table S14. Associations between nature exposures at 300 or 1000 m buffer and incidence of mental disorders among people with diabetes who had lived at the current address more than 10 years before baseline. Table S15. Associations between nature exposures at 300 or 1000 m buffer and incidence of mental disorders among people with diabetes after excluding participants with mental disorders events within 1 years of follow-up. Table S16. Associations between nature exposures at 300 or 1000 m buffer and incidence of mental disorders among people with diabetes after excluding the participants with missing data on covariates. Table S17. Crude hazard ratios and 95% confidence interval of incident mental disorders associated with nature exposures at 300 or 1000 m buffer among people with diabetes. Table S18. Associations between nature exposures at 300 m buffer and incidence of mental disorders among people with diabetes with adjustment air pollutants. Table S19. Associations between nature exposures at 1000 m buffer and incidence of mental disorders among people with diabetes with adjustment air pollutants.

## Data Availability

Researchers can request the data used for the analysis from the UK Biobank (http://www.ukbiobank.ac.uk/).

## Abbreviations

CEH: Centre for Ecology and Hydrology
CIs: Confidence intervals
CVD: Cardiovascular disease
GLUD: Generalized Land Use Database
HRs: Hazard ratios
ICD-10: International Classification of Diseases 10th revision
LCM: Land Cover Map
LSOAs: Lower-layer super output areas
MET: Metabolic equivalent task
NHS: National Health Service
NO_x_: Nitrogen oxides
NO_2_: Nitrogen dioxide
PAF: Population attributable fraction
PM: Particulate matter
PM_2.5_: Particulate matter with aerodynamic diameter ≤2.5 µm
PM_10_: Particulate matter with aerodynamic diameter ≤10 µm
RCS: Restricted cubic spline
SD: Standard deviations
SMI: Severe mental illnesses
T2D: Type 2 diabetes
UKB: UK Biobank
