# Supplementary material for: Green sanctuaries: residential green and garden space and the natural environment mitigate mental disorders risk of diabetic patients
Source: BMC Med. 2025 Jan 21;23:31. doi: 10.1186/s12916-025-03864-y (PMC11752615; doi:10.1186/s12916-025-03864-y)
Supplement: Supplementary file 1 — Additional file 1: Fig. S1. Flow chart of the inclusion of the participants. Fig. S2. The verification of the proportional hazard assumption in fully adjusted model examined the relationship between green space and incident mental disorders. Fig. S3. The verification of the proportional hazard assumption in fully adjusted model examined the relationship between garden space and incident mental disorders. Fig. S4. The verification of the proportional hazard assumption in fully adjusted model examined the relationship between natural environment and incident mental disorders. Fig. S5. Distribution of residential green space, garden space, and natural environment at baseline. Table S1. ICD-10 codes assigned for mental disorders. Table S2. Definitions for covariates. Table S3. Mental disorders events from 39,397 participants with diabetes in the UK Biobank. Table S4. Distribution of residential green space, garden space, and natural environment at baseline. Table S5. Associations between nature exposures at 300 or 1000 m buffer and incidence of depressive disorders among people with diabetes. Table S6. Associations between nature exposures at 300 or 1000 m buffer and incidence of anxiety disorders among people with diabetes. Table S7. Correlations between nature exposures (Spearman correlation coefficients). Table S8. Mediation analysis in associations of nature exposures at 300 m and 1,000 m buffer with incidence of mental disorders among participants with diabetes. Table S9. Associations between exposure to green space at 300 m buffer and incidence of mental disorders among people with diabetes in stratified analyses. Table S10. Associations between exposure to natural environment at 300 m buffer and incidence of mental disorders among people with diabetes in stratified analyses. Table S11. Associations between exposure to garden space at 300 m buffer and incidence of mental disorders among people with diabetes in stratified analyses. Table S12. Associations between n [file 12916_2025_3864_MOESM1_ESM.docx]

**Additional file 1**

**Green sanctuaries: residential green and garden space and the natural environment mitigate mental disorders risk of diabetic patients**

Erxu Xue^1,2,5†^, Jianhui Zhao^2†^*, Jingyu Ye^3^, Jingjie Wu^1^, Dandan Chen^1^, Jing Shao^4, 5^, Xue Li^2^*, Zhihong Ye^1^*

^1^ Nursing Department, Sir Run Run Shaw Hospital, Zhejiang University School of Medicine, Hangzhou, China.

^2^ Department of Big Data in Health Science School of Public Health and The Second Affiliated Hospital, Zhejiang University School of Medicine, Hangzhou, China.

^3^ Department of Epidemiology, School of Public Health, Harbin Medical University, Harbin, China.

^4^ Department of Nursing, the Fourth Affiliated Hospital of School of Medicine, and International School of Medicine, International Institutes of Medicine, Zhejiang University, Yiwu, China.

^5^ Institute of Nursing Research, School of Medicine Zhejiang University, Hangzhou, China.

^†^ Erxu Xue and Jianhui Zhao contributed equally and share first authorship.

* **Correspondence:**

Zhihong Ye (3192005@zju.edu.cn), Xue Li (xueli157@zju.edu.cn), and Jianhui Zhao (jianhui_zhao@zju.edu.cn)

**Electronic supplementary material**

**Fig. S1.** Flow chart of the inclusion of the participants.

**Fig. S2.** The verification of the proportional hazard assumption in fully adjusted model examined the relationship between green space and incident mental disorders.

**Fig. S3.** The verification of the proportional hazard assumption in fully adjusted model examined the relationship between garden space and incident mental disorders.

**Fig. S4.** The verification of the proportional hazard assumption in fully adjusted model examined the relationship between natural environment and incident mental disorders.

**Fig. S5.** Distribution of residential green space, garden space, and natural environment at baseline.

**Table S1.** ICD-10 codes assigned for mental disorders.

**Table S2.** Definitions for covariates.

**Table S3.** Mental disorders events from 39,397 participants with diabetes in the UK Biobank.

**Table S4.** Distribution of residential green space, garden space , and natural environment at baseline.

**Table S5.** Associations between nature exposures at 300 or 1000 m buffer and incidence of depressive disorders among people with diabetes.

**Table S6.** Associations between nature exposures at 300 or 1000 m buffer and incidence of anxiety disorders among people with diabetes.

**Table S7.** Correlations between nature exposures (Spearman correlation coefficients).

**Table S8.** Mediation analysis in associations of nature exposures at 300 m and 1,000 m buffer with incidence of mental disorders among participants with diabetes.

**Table S9.** Associations between exposure to green space at 300 m buffer and incidence of mental disorders among people with diabetes in stratified analyses.

**Table S10.** Associations between exposure to natural environment at 300 m buffer and incidence of mental disorders among people with diabetes in stratified analyses.

**Table S11.** Associations between exposure to garden space at 300 m buffer and incidence of mental disorders among people with diabetes in stratified analyses.

**Table S12.** Associations between nature exposures at 300 or 1000 m buffer and incidence of mental disorders among people with type 2 diabetes (N = 37,519).

**Table S13.** Associations between nature exposures at 300 or 1000 m buffer and incidence of mental disorders among people without diabetes (N = 410,338).

**Table S14.** Associations between nature exposures at 300 or 1000 m buffer and incidence of mental disorders among people with diabetes who had lived at the current address more than 10 years before baseline (N = 27,327).

**Table S15.** Associations between nature exposures at 300 or 1000 m buffer and incidence of mental disorders among people with diabetes after excluding participants with mental disorders events within 1 years of follow-up (N = 38,833).

**Table S16.** Associations between nature exposures at 300 or 1000 m buffer and incidence of mental disorders among people with diabetes after excluding the participants with missing data on covariates (N = 19,679).

**Table S17.** Crude hazard ratios and 95 % confidence interval of incident mental disorders associated with nature exposures at 300 or 1000 m buffer among people with diabetes (N = 39,397).

**Table S18.** Associations between nature exposures at 300 m buffer and incidence of mental disorders among people with diabetes with adjustment air pollutants (N = 38,983).

**Table S19.** Associations between nature exposures at 1000 m buffer and incidence of mental disorders among people with diabetes with adjustment air pollutants (N = 38,983).


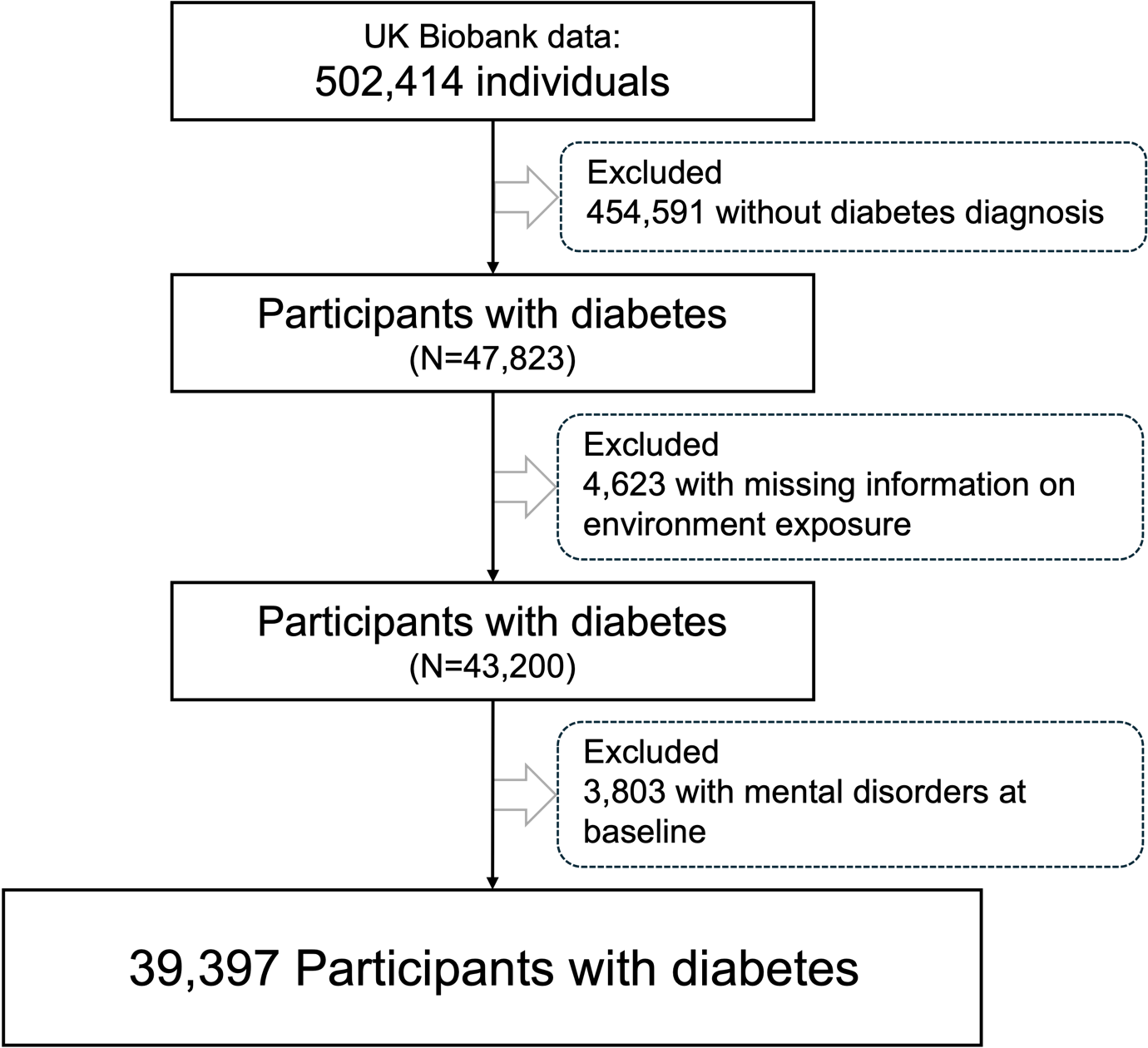


Fig. S1. Flow chart of the inclusion of the participants.

Fig. S2. The verification of the proportional hazard assumption in fully adjusted model examined the relationship between green space and incident mental disorders.

**Fig. S3.** The verification of the proportional hazard assumption in fully adjusted model examined the relationship between garden space and incident mental disorders.

**Fig. S4.** The verification of the proportional hazard assumption in fully adjusted model examined the relationship between natural environment and incident mental disorders.

**Fig. S5.** Distribution of residential green space, garden space, and natural environment at baseline.

**Table S1.** ICD-10 codes assigned for mental disorders. **^a^**

| **Diagnosis** | **Detailed diagnostic code** |
| --- | --- |
| Depressive disorder | F320, F321, F322, F323, F328, F329, F330, F331, F332, F333, F334, F338, F339 |
| Anxiety disorder | F400, F401, F402, F408, F409, F410, F411, F412, F413, F418, F419 |
| Schizophrenia | F200, F201, F202, F203, F204, F205, F206, F208, F209 |
| Schizotypal disorder | F21 |
| Persistent delusional disorders | F220, F228, F229 |
| Acute polymorphic psychotic disorder without symptoms of schizophrenia | F230 |
| Induced delusional disorder | F24 |
| Schizoaffective disorders | F250, F251, F252, F258, F259 |
| Other nonorganic psychotic disorders | F28 |
| Unspecified nonorganic psychosis | F29 |
| Manic episode | F300, F301, F302, F308, F309 |
| Bipolar affective disorder | F310, F311, F312, F313, F314, F315, F316, F317, F318, F319 |
| Post-traumatic stress disorder | F431 |

^a^ ICD-10 was used for inpatient data.

**Abbreviations:** ICD, International Classification of Diseases.

**Table S2.** Definitions for covariates.

|  | **Definition** | **Field code** |
| --- | --- | --- |
| Age | Age based on the date of birth and date of attending an initial assessment center and refers to the age of the participant on the day they attended an Initial Assessment Centre (variable handling: **<65** or **>=65** years old) | 21003 |
| Sex | A mixture of the sex the National Health Service had recorded for the participant and self-reported sex. Self-reported sex will be used to update the sex information recorded in the central registry. (variable handling: categorical variable **“Female”**, **“Male”**) | 31 |
| Ethnicity | Self-reported: “What is your ethnic background”. We classified the variable into: **White** and **Others** (Mixed, Asian or Asian British, Black or Black British, Chinese, and other ethnic groups) because the number of non-white ethnic backgrounds was too small | 21000 |
| Household income | Self-reported: "What is the average total income before tax received by your HOUSEHOLD?". Building on prior research, we classified the variable into **high: ≥ £52,000**, **low: < £52 000** | 738 |
| Current employment status | Self-reported: "Which of the following describes your current situation? (You can select more than one answer)". We classified the variable into **current employed** or **not** | 6142 |
| Education level | Self-reported: “Which of the following qualifications do you have” We classified the variable into College (College or University degree) and Below college (A levels/AS levels or equivalent, O levels/GCSEs or equivalent, CSEs or equivalent, NVQ or HND or HNC or equivalent, other professional qualifications eg: nursing, teaching, and none of the above) (classified into: **College/university degree** or **Other qualifications**) | 6138 |
| Smoking status | Self-reported: current/past smoking status of the participant. We classified the variable handlings into **Never**, **former** and **Current smoker** | 20116 |
| Alcohol consumption | Participants self-reported the number of alcohol units (10 ml of pure ethanol) consumed, in “units per week” (for frequent drinkers) or “units per month” (for less frequent drinkers), across several beverage categories (red wine, white wine/champagne, beer/cider, spirits, fortified wine, and “other”). To calculate alcohol consumption as per guidelines, multiply the volume by the alcohol content in percent and divide by drink-equivalent; then convert to grams:  1 drink equivalent described as containing 14g of pure alcohol.  125ml wine = 0.85 drink-equivalents,  4% ABV pint beer = 1.28 drink-equivalents,  25ml spirits = 0.57 drink-equivalents,  50ml fortified wine = 0.56 drink-equivalents,  None to moderate level of alcohol consumption was defined as 0-14 g/d for  women and 0-28 g/d for men according to US dietary guidelines, which is defined as a heavy level (variable handling: categorical variable “**None to moderate**”, “**Heavy**”) | 1568, 1578, 1588, 1598,  1608 |
| Physical activity | Evaluated by metabolic equivalent task [MET], According to the median number of total exercise days performing walking, moderate, and vigorous activity, they were divided into two groups : **high** and **low** | 22033 |
| Healthy  diet pattern | The evaluation for the diet pattern was based on five dietary factors: vegetable intake of at least four tablespoons each day (median); fruits intake at least three pieces each day (median); fish intake at least twice each week (median); unprocessed red meat intake no more than twice each week (median); and processed meat intake no more than two each week (median). Each point was given for each favorable diet factor, with the total diet score ranging from 0 to 5, and a **healthy diet** pattern was defined as a healthy sleep score ≥ 4 points | 1289, 1299, 1309, 1319, 1329, 1339, 1369, 1379, 1389, 1349 |
| Healthy  sleep pattern | The sleep pattern is evaluated by a healthy sleep score calculated from five factors: insomnia, sleep duration, chronotype, daytime sleepiness, and snoring. Healthy sleep factors were defined as “never/rarely or sometimes insomnia symptoms”, “sleep 7-8 h per day”, “no self-reported snoring”, and “no excessive daytime sleepiness” (never/rarely or sometimes).  Each sleep factor is coded 1 point if meeting the healthy criterion and 0 if not. The healthy sleep score ranged from 0-5, and a **healthy sleep** pattern was defined as a healthy sleep score ≥ 4 points | 1200, 1180, 1160, 1220, 1210 |

**Table S3.** Mental disorders events from 39,397 participants with diabetes in the UK Biobank.

| **Mental disorders** | **Number of cases** |
| --- | --- |
| **Total** | **4,513** |
| Depressive disorder | 2,952 |
| Recurrent depressive disorder | 12 |
| Anxiety | 1,209 |
| Schizophrenia | 113 |
| Schizotypal disorder | 1 |
| Persistent delusional disorders | 52 |
| Acute polymorphic psychotic disorder without symptoms of schizophrenia | 12 |
| Schizoaffective disorders | 6 |
| Unspecified nonorganic psychosis | 23 |
| Manic episode | 16 |
| Bipolar affective disorder | 98 |
| Post-traumatic stress disorder | 19 |

**Table S4.** Distribution of residential green space, garden space , and natural environment at baseline.

| **Exposures** | **Mean** | **Standard Deviation** | **25th Percentile** | **Median** | **75th Percentile** | **Inter-quartile Range** |
| --- | --- | --- | --- | --- | --- | --- |
| **Green space (%)** |  |  |  |  |  |  |
| 300m buffer | 33.21 | 21.41 | 17.06 | 27.99 | 44.71 | 27.65 |
| 1000m buffer | 42.91 | 20.50 | 26.38 | 39.33 | 42.91 | 30.23 |
| **Natural environment (%)** |  |  |  |  |  |  |
| 300m buffer | 23.99 | 23.65 | 5.13 | 17.19 | 36.16 | 31.03 |
| 1000m buffer | 37.88 | 24.74 | 17.46 | 33.68 | 54.72 | 37.26 |
| **Garden space (%)** |  |  |  |  |  |  |
| 300m buffer | 30.96 | 14.11 | 20.73 | 31.45 | 41.32 | 20.58 |
| 1000m buffer | 24.31 | 10.82 | 16.72 | 24.14 | 31.75 | 15.03 |

**Table S5.** Associations between nature exposures at 300 or 1000 m buffer and incidence of depressive disorders among people with diabetes.

|  | **300 m buffer** | | | | **1000 m buffer** | | | |
| --- | --- | --- | --- | --- | --- | --- | --- | --- |
|  | **N** | **Cases/Person-years** | **HR (95% CI)** | **P-Value** | **N** | **Cases/Person-years** | **HR (95% CI)** | **P-Value** |
| **Green space** |  |  |  |  |  |  |  |  |
| Tertile 1 | 12,556 | 944/95,346 | Reference |  | 12,521 | 1,037/95,136 | Reference |  |
| Tertile 2 | 12,631 | 1,065/96,066 | 1.09 (1.00-1.19) | 0.047 | 12,638 | 1,009/96,112 | 0.95 (0.87-1.04) | 0.246 |
| Tertile 3 | 12,649 | 943/96,794 | 1.00 (0.91-1.09) | 0.923 | 12,677 | 906/96,959 | 0.87 (0.80-0.96) | 0.004 |
| P for trend | - | - | - | 0.922 | - | - | - | 0.004 |
| **Natural environment** |  |  |  |  |  |  |  |  |
| Tertile 1 | 12,683 | 1,052/96,139 | Reference |  | 12,543 | 1,067/95,309 | Reference |  |
| Tertile 2 | 12,545 | 976/95,200 | 0.94 (0.86-1.02) | 0.157 | 12,614 | 992/95,982 | 0.92 (0.84-1.01) | 0.068 |
| Tertile 3 | 12,608 | 924/96,868 | 0.90 (0.82-0.98) | 0.016 | 12,679 | 893/96,916 | 0.85 (0.78-0.93) | 0.001 |
| P for trend | - | - | - | 0.016 | - | - | - | 0.001 |
| **Garden space** |  |  |  |  |  |  |  |  |
| Tertile 1 | 12,565 | 1,032/95,199 | Reference |  | 12,593 | 1,026/96,041 | Reference |  |
| Tertile 2 | 12,618 | 1,047/96,816 | 1.01 (0.93-1.11) | 0.741 | 12,622 | 981/95,957 | 0.96 (0.88-1.05) | 0.421 |
| Tertile 3 | 12,653 | 873/96,192 | 0.87 (0.80-0.95) | 0.003 | 12,621 | 945/96,209 | 0.95 (0.87-1.04) | 0.254 |
| P for trend | - | - | - | 0.004 | - | - | - | 0.252 |

The analysis was performed in Model 2 (adjusted with for age, sex, ethnicity, household income, education, employed, smoking, alcohol consumption, physical activity (MET), healthy diet pattern, and healthy sleep pattern).

**Abbreviations:** CI, confidence interval; HR, hazard ratio; MET, metabolic equivalent of task.

**Table S6.** Associations between nature exposures at 300 or 1000 m buffer and incidence of anxiety disorders among people with diabetes.

|  | **300 m buffer** | | | | **1000 m buffer** | | | |
| --- | --- | --- | --- | --- | --- | --- | --- | --- |
|  | **N** | **Cases/Person-years** | **HR (95% CI)** | **P-Value** | **N** | **Cases/Person-years** | **HR (95% CI)** | **P-Value** |
| **Green space** |  |  |  |  |  |  |  |  |
| Tertile 1 | 12,048 | 436/92,563 | Reference |  | 11,934 | 450/91,980 | Reference |  |
| Tertile 2 | 11,962 | 396/92,515 | 0.90 (0.79-1.04) | 0.150 | 12,013 | 384/92,850 | 0.85 (0.74-0.97) | 0.020 |
| Tertile 3 | 12,083 | 377/93,694 | 0.87 (0.75-1.00) | 0.047 | 12,146 | 375/93,942 | 0.83 (0.72-0.95) | 0.008 |
| P for trend | - | - | - | 0.046 | - | - | - | 0.008 |
| **Natural environment** |  |  |  |  |  |  |  |  |
| Tertile 1 | 12,069 | 438/92,663 | Reference |  | 11,913 | 437/91,888 | Reference |  |
| Tertile 2 | 11,958 | 389/92,057 | 0.90 (0.79-1.04) | 0.153 | 12,022 | 400/92,873 | 0.91 (0.79-1.04) | 0.178 |
| Tertile 3 | 12,066 | 382/94,051 | 0.87 (0.76-1.00) | 0.055 | 12,158 | 372/94,010 | 0.85 (0.74-0.98) | 0.023 |
| P for trend | - | - | - | 0.054 | - | - | - | 0.023 |
| **Garden space** |  |  |  |  |  |  |  |  |
| Tertile 1 | 11,955 | 422/92,086 | Reference |  | 11,980 | 413/92,661 | Reference |  |
| Tertile 2 | 11,972 | 401/93,233 | 0.95 (0.83-1.09) | 0.442 | 12,049 | 408/92,977 | 0.99 (0.87-1.14) | 0.922 |
| Tertile 3 | 12,166 | 386/93,453 | 0.92 (0.80-1.05) | 0.209 | 12,064 | 388/93,134 | 0.94 (0.82-1.08) | 0.411 |
| P for trend | - | - | - | 0.209 | - | - | - | 0.413 |

The analysis was performed in Model 2 (adjusted with for age, sex, ethnicity, household income, education, employed, smoking, alcohol consumption, physical activity (MET), healthy diet pattern, and healthy sleep pattern).

**Abbreviations:** CI, confidence interval; HR, hazard ratio; MET, metabolic equivalent of task.

**Table S7.** Correlations between nature exposures (Spearman correlation coefficients).

|  | Green space (300 m) | Green space (1000 m) | Natural environment (300 m) | Natural environment (1000 m) | Garden space (300 m) | Garden space (1000 m) | NO_2_ | NO_x_ | PM_10_ | PM_2.5_ |
| --- | --- | --- | --- | --- | --- | --- | --- | --- | --- | --- |
| Green space (300 m) | 1 |  |  |  |  |  |  |  |  |  |
| Green space (1000 m) | 0.832 | 1 |  |  |  |  |  |  |  |  |
| Natural environment (300 m) | 0.875 | 0.744 | 1 |  |  |  |  |  |  |  |
| Natural environment (1000 m) | 0.799 | 0.964 | 0.770 | 1 |  |  |  |  |  |  |
| Garden space (300 m) | -0.684 | -0.417 | -0.535 | -0.355 | 1 |  |  |  |  |  |
| Garden space (1000 m) | -0.638 | -0.681 | -0.514 | -0.606 | 0.784 | 1 |  |  |  |  |
| NO_2_ ^*^ | -0.608 | -0.718 | -0.606 | -0.742 | 0.112 | 0.275 | 1 |  |  |  |
| NO_x_ ^*^ | -0.498 | -0.534 | -0.526 | -0.559 | 0.043 | 0.132 | 0.922 | 1 |  |  |
| PM_10_ ^*^ | -0.381 | -0.355 | -0.415 | -0.375 | 0.161 | 0.172 | 0.500 | 0.506 | 1 |  |
| PM_2.5_ ^*^ | -0.599 | -0.616 | -0.617 | -0.650 | 0.086 | 0.165 | 0.857 | 0.837 | 0.520 | 1 |

* Annual average concentration of NO_2_, NO_x_, PM_10_ and PM_2.5_ in 2010 at each participant’s residential address.

**Abbreviations:** NO_2_, nitrogen dioxide; NO_x_, nitrogen oxides; PM_10_, particulate matter (PM) with aerodynamic diameter ≤10 µm; PM_2.5_, PM with aerodynamic diameter < 2.5 µm.

**Table S8.** Mediation analysis in associations of nature exposures at 300 m and 1,000 m buffer with incidence of mental disorders among participants with diabetes.

|  | **NO_2_** | | **NO_x_** | | **PM_10_** | | **PM_2.5_** | |
| --- | --- | --- | --- | --- | --- | --- | --- | --- |
|  | **Mediation**  **proportion** | **P value** | **Mediation**  **proportion** | **P value** | **Mediation**  **proportion** | **P value** | **Mediation**  **proportion** | **P value** |
|  |  |  |  |  |  |  |  |  |
| **300 m buffer** |  |  |  |  |  |  |  |  |
| **Green space** |  |  |  |  |  |  |  |  |
| Percentage mediated | 77.1% (24.4%-225.6) | 0.006 | 44.3% (9.6%-128.1%) | 0.008 | 8.8% (-20.0%-46.6%) | 0.488 | 92.1% (38.1%-274.2%) | 0.010 |
| **Natural environment** |  |  |  |  |  |  |  |  |
| Percentage mediated | 48.3% (10.6%-115.2%) | 0.012 | 29.2% (2.2%-77.1%) | 0.034 | 2.3% (-24.6%-27.6%) | 0.886 | 62.4% (24.1%-158.1%) | <0.001 |
| **1,000m buffer** |  |  |  |  |  |  |  |  |
| **Green space** |  |  |  |  |  |  |  |  |
| Percentage mediated | 37.3% (-6.1%-93.1%) | 0.084 | 20.7% (-3.1%-52.0%) | 0.084 | 2.1% (-15.9%-18.8%) | 0.858 | 45.8% (15.5%-98.0%) | 0.004 |
| **Natural environment** |  |  |  |  |  |  |  |  |
| Percentage mediated | 22.2% (-18.7-69.8%) | 0.252 | 14.3% (-11.9%-40.1%) | 0.290 | -0.3% (-16.7%-14.4%) | 0.954 | 35.8% (5.6%-80.1%) | 0.026 |

The analysis was performed in Model 2 (adjusted with for age, sex, ethnicity, household income, education, employed, smoking, alcohol consumption, physical activity (MET), healthy diet pattern, and healthy sleep pattern).

**Abbreviations:** MET, metabolic equivalent of task; NO_2_, nitrogen dioxide; NO_x_, nitrogen oxides; PM_10_, particulate matter (PM) with aerodynamic diameter ≤10 µm; PM_2.5_, PM with aerodynamic diameter < 2.5 µm.

**Table S9.** Associations between exposure to green space at 300 m buffer and incidence of mental disorders among people with diabetes in stratified analyses.

| **Subgroup** | **No. case/Total** | **Group** | | | | | **P for interaction** |
| --- | --- | --- | --- | --- | --- | --- | --- |
|  |  | **Tertile 1** | **Tertile 2** | **P value** | **Tertile 3** | **P value** |  |
| Age (years) |  |  |  |  |  |  | 0.058 |
| < 65 | 3,247/27,464 | 1.00 [Reference] | 1.03 (0.95-1.12) | 0.531 | 0.92 (0.84-1.00) | 0.056 |  |
| ≥ 65 | 1,266/11,933 | 1.00 [Reference] | 0.95 (0.83-1.09) | 0.455 | 1.01 (0.88-1.16) | 0.886 |  |
| Sex |  |  |  |  |  |  | 0.315 |
| Female | 2,272/15,803 | 1.00 [Reference] | 1.06 (0.96-1.17) | 0.261 | 0.97 (0.87-1.08) | 0.569 |  |
| Male | 2,241/23,594 | 1.00 [Reference] | 0.95 (0.86-1.05) | 0.354 | 0.92 (0.83-1.02) | 0.109 |  |
| Education |  |  |  |  |  |  | 0.374 |
| Non-college | 3,655/30,880 | 1.00 [Reference] | 1.01 (0.93-1.09) | 0.839 | 0.96 (0.89-1.05) | 0.370 |  |
| College | 858/8,517 | 1.00 [Reference] | 0.99 (0.84-1.16) | 0.898 | 0.87 (0.73-1.02) | 0.091 |  |
| Household income |  |  |  |  |  |  | 0.354 |
| Low | 3,232/27,215 | 1.00 [Reference] | 1.05 (0.96-1.14) | 0.272 | 0.96 (0.88-1.04) | 0.316 |  |
| High | 318/4,577 | 1.00 [Reference] | 0.81 (0.61-1.08) | 0.152 | 0.87 (0.67-1.13) | 0.289 |  |
| Employment status |  |  |  |  |  |  | 0.990 |
| Currently unemployed | 3,061/22,686 | 1.00 [Reference] | 1.01 (0.93-1.10) | 0.809 | 0.95 (0.87-1.04) | 0.259 |  |
| Currently employed | 1,452/16,711 | 1.00 [Reference] | 1.00 (0.88-1.13) | 0.996 | 0.94 (0.83-1.07) | 0.336 |  |
| Smoking status |  |  |  |  |  |  | 0.150 |
| Never | 1,921/18,271 | 1.00 [Reference] | 1.05 (0.94-1.17) | 0.411 | 1.04 (0.93-1.17) | 0.458 |  |
| Former | 1,856/16,367 | 1.00 [Reference] | 0.96 (0.86-1.07) | 0.457 | 0.86 (0.77-0.96) | 0.009 |  |
| Current | 736/4,759 | 1.00 [Reference] | 1.01 (0.85-1.19) | 0.951 | 0.92 (0.77-1.11) | 0.380 |  |
| Alcohol consumption |  |  |  |  |  |  | 0.037 |
| None to moderate | 3,751/31,685 | 1.00 [Reference] | 0.99 (0.92-1.07) | 0.869 | 0.97 (0.89-1.05) | 0.443 |  |
| Heavy | 762/7,712 | 1.00 [Reference] | 1.06 (0.89-1.26) | 0.525 | 0.83 (0.70-0.99) | 0.044 |  |
| Physical activity (MET) |  |  |  |  |  |  | 0.982 |
| Low | 1,841/15,453 | 1.00 [Reference] | 0.98 (0.88-1.10) | 0.757 | 0.95 (0.85-1.06) | 0.379 |  |
| High | 1,221/12,515 | 1.00 [Reference] | 1.03 (0.89-1.17) | 0.722 | 0.95 (0.82-1.09) | 0.432 |  |
| Healthy diet |  |  |  |  |  |  | 0.446 |
| Unhealthy | 2,878/25,764 | 1.00 [Reference] | 0.99 (0.90-1.08) | 0.782 | 0.92 (0.84-1.00) | 0.061 |  |
| Healthy | 1,234/10,714 | 1.00 [Reference] | 1.02 (0.89-1.16) | 0.804 | 0.99 (0.86-1.14) | 0.925 |  |
| Healthy sleep |  |  |  |  |  |  | 0.743 |
| Unhealthy | 2,191/16,287 | 1.00 [Reference] | 0.99 (0.90-1.10) | 0.871 | 0.95 (0.85-1.05) | 0.295 |  |
| Healthy | 1,264/14,761 | 1.00 [Reference] | 1.00 (0.87-1.14) | 0.950 | 0.97 (0.85-1.11) | 0.678 |  |

The analysis was performed in Model 2 (adjusted with for age, sex, ethnicity, household income, education, employed, smoking, alcohol consumption, physical activity (MET), healthy diet pattern, and healthy sleep pattern).

**Abbreviations:** MET, metabolic equivalent of task.

**Table S10.** Associations between exposure to natural environment at 300 m buffer and incidence of mental disorders among people with diabetes in stratified analyses.

| **Subgroup** | **No. case/Total** | **Group** | | | | | **P for interaction** |
| --- | --- | --- | --- | --- | --- | --- | --- |
|  |  | **Tertile 1** | **Tertile 2** | **P value** | **Tertile 3** | **P value** |  |
| Age (years) |  |  |  |  |  |  | 0.075 |
| < 65 | 3,247/27,464 | 1.00 [Reference] | 0.94 (0.86-1.02) | 0.114 | 0.85 (0.78-0.92) | <0.001 |  |
| ≥ 65 | 1,266/11,933 | 1.00 [Reference] | 0.91 (0.79-1.04) | 0.158 | 0.96 (0.84-1.10) | 0.594 |  |
| Sex |  |  |  |  |  |  | 0.136 |
| Female | 2,272/15,803 | 1.00 [Reference] | 0.97 (0.88-1.07) | 0.552 | 0.92 (0.83-1.02) | 0.119 |  |
| Male | 2,241/23,594 | 1.00 [Reference] | 0.88 (0.80-0.97) | 0.013 | 0.84 (0.75-0.93) | 0.001 |  |
| Education |  |  |  |  |  |  | 0.467 |
| Non-college | 3,655/30,880 | 1.00 [Reference] | 0.93 (0.86-1.00) | 0.053 | 0.89 (0.82-0.97) | 0.006 |  |
| College | 858/8,517 | 1.00 [Reference] | 0.93 (0.79-1.10) | 0.390 | 0.82 (0.70-0.97) | 0.023 |  |
| Household income |  |  |  |  |  |  | 0.942 |
| Low | 3,232/27,215 | 1.00 [Reference] | 0.93 (0.85-1.01) | 0.078 | 0.87 (0.80-0.95) | 0.002 |  |
| High | 318/4,577 | 1.00 [Reference] | 0.91 (0.68-1.21) | 0.508 | 0.95 (0.73-1.23) | 0.683 |  |
| Employment status |  |  |  |  |  |  | 0.437 |
| Currently unemployed | 3,061/22,686 | 1.00 [Reference] | 0.92 (0.84-1.00) | 0.054 | 0.86 (0.79-0.94) | 0.001 |  |
| Currently employed | 1,452/16,711 | 1.00 [Reference] | 0.94 (0.83-1.07) | 0.339 | 0.93 (0.82-1.05) | 0.253 |  |
| Smoking status |  |  |  |  |  |  | 0.030 |
| Never | 1,921/18,271 | 1.00 [Reference] | 0.99 (0.89-1.11) | 0.907 | 1.00 (0.89-1.12) | 0.993 |  |
| Former | 1,856/16,367 | 1.00 [Reference] | 0.88 (0.79-0.98) | 0.023 | 0.79 (0.71-0.89) | <0.001 |  |
| Current | 736/4,759 | 1.00 [Reference] | 0.88 (0.74-1.05) | 0.166 | 0.82 (0.68-0.98) | 0.027 |  |
| Alcohol consumption |  |  |  |  |  |  | 0.011 |
| None to moderate | 3,751/31,685 | 1.00 [Reference] | 0.92 (0.85-1.00) | 0.037 | 0.91 (0.84-0.99) | 0.020 |  |
| Heavy | 762/7,712 | 1.00 [Reference] | 0.94 (0.79-1.12) | 0.497 | 0.73 (0.61-0.88) | 0.001 |  |
| Physical activity (MET) |  |  |  |  |  |  | 0.374 |
| Low | 1,841/15,453 | 1.00 [Reference] | 0.87 (0.78-0.98) | 0.017 | 0.85 (0.76-0.95) | 0.005 |  |
| High | 1,221/12,515 | 1.00 [Reference] | 0.96 (0.84-1.10) | 0.581 | 0.85 (0.74-0.98) | 0.022 |  |
| Healthy diet |  |  |  |  |  |  | 0.103 |
| Unhealthy | 2,878/25,764 | 1.00 [Reference] | 0.91 (0.84-1.00) | 0.044 | 0.84 (0.77-0.92) | <0.001 |  |
| Healthy | 1,234/10,714 | 1.00 [Reference] | 0.94 (0.82-1.08) | 0.396 | 0.93 (0.81-1.07) | 0.291 |  |
| Healthy sleep |  |  |  |  |  |  | 0.322 |
| Unhealthy | 2,191/16,287 | 1.00 [Reference] | 0.91 (0.82-1.00) | 0.058 | 0.86 (0.78-0.95) | 0.004 |  |
| Healthy | 1,264/14,761 | 1.00 [Reference] | 0.97 (0.84-1.11) | 0.638 | 0.96 (0.84-1.10) | 0.604 |  |

The analysis was performed in Model 2 (adjusted with for age, sex, ethnicity, household income, education, employed, smoking, alcohol consumption, physical activity (MET), healthy diet pattern, and healthy sleep pattern).

**Abbreviations**: MET, metabolic equivalent of task.

**Table S11.** Associations between exposure to garden space at 300 m buffer and incidence of mental disorders among people with diabetes in stratified analyses.

| **Subgroup** | **No. case/Total** | **Group** | | | | | **P for interaction** |
| --- | --- | --- | --- | --- | --- | --- | --- |
|  |  | **Tertile 1** | **Tertile 2** | **P value** | **Tertile 3** | **P value** |  |
| Age (years) |  |  |  |  |  |  | 0.104 |
| < 65 | 3,247/27,464 | 1.00 [Reference] | 1.02 (0.94-1.11) | 0.599 | 0.88 (0.81-0.96) | 0.003 |  |
| ≥ 65 | 1,266/11,933 | 1.00 [Reference] | 0.86 (0.75-0.99) | 0.029 | 0.85 (0.75-0.98) | 0.021 |  |
| Sex |  |  |  |  |  |  | 0.158 |
| Female | 2,272/15,803 | 1.00 [Reference] | 1.04 (0.95-1.15) | 0.396 | 0.90 (0.81-1.00) | 0.044 |  |
| Male | 2,241/23,594 | 1.00 [Reference] | 0.91 (0.83-1.01) | 0.073 | 0.85 (0.76-0.94) | 0.002 |  |
| Education |  |  |  |  |  |  | 0.442 |
| Non-college | 3,655/30,880 | 1.00 [Reference] | 0.96 (0.89-1.04) | 0.296 | 0.87 (0.80-0.94) | 0.001 |  |
| College | 858/8,517 | 1.00 [Reference] | 1.05 (0.90-1.24) | 0.518 | 0.90 (0.76-1.06) | 0.221 |  |
| Household income |  |  |  |  |  |  | 0.130 |
| Low | 3,232/27,215 | 1.00 [Reference] | 1.02 (0.94-1.11) | 0.605 | 0.88 (0.81-0.96) | 0.005 |  |
| High | 318/4,577 | 1.00 [Reference] | 0.99 (0.75-1.30) | 0.917 | 0.96 (0.73-1.25) | 0.749 |  |
| Employment status |  |  |  |  |  |  | 0.055 |
| Currently unemployed | 3,061/22,686 | 1.00 [Reference] | 0.93 (0.85-1.01) | 0.080 | 0.85 (0.77-0.92) | <0.001 |  |
| Currently employed | 1,452/16,711 | 1.00 [Reference] | 1.10 (0.97-1.24) | 0.150 | 0.94 (0.83-1.07) | 0.353 |  |
| Smoking status |  |  |  |  |  |  | 0.243 |
| Never | 1,921/18,271 | 1.00 [Reference] | 0.99 (0.89-1.10) | 0.861 | 0.87 (0.78-0.97) | 0.014 |  |
| Former | 1,856/16,367 | 1.00 [Reference] | 0.96 (0.86-1.08) | 0.511 | 0.92 (0.82-1.03) | 0.141 |  |
| Current | 736/4,759 | 1.00 [Reference] | 0.97 (0.82-1.14) | 0.691 | 0.75 (0.62-0.90) | 0.002 |  |
| Alcohol consumption |  |  |  |  |  |  | 0.568 |
| None to moderate | 3,751/31,685 | 1.00 [Reference] | 0.96 (0.89-1.04) | 0.342 | 0.86 (0.80-0.94) | <0.001 |  |
| Heavy | 762/7,712 | 1.00 [Reference] | 1.04 (0.88-1.23) | 0.650 | 0.89 (0.75-1.07) | 0.221 |  |
| Physical activity (MET) |  |  |  |  |  |  | 0.267 |
| Low | 1,841/15,453 | 1.00 [Reference] | 0.95 (0.86-1.06) | 0.390 | 0.80 (0.72-0.90) | <0.001 |  |
| High | 1,221/12,515 | 1.00 [Reference] | 1.05 (0.91-1.20) | 0.514 | 0.90 (0.78-1.03) | 0.137 |  |
| Healthy diet |  |  |  |  |  |  | 0.929 |
| Unhealthy | 2,878/25,764 | 1.00 [Reference] | 0.98 (0.90-1.07) | 0.606 | 0.86 (0.79-0.94) | 0.001 |  |
| Healthy | 1,234/10,714 | 1.00 [Reference] | 0.99 (0.86-1.13) | 0.844 | 0.90 (0.78-1.03) | 0.135 |  |
| Healthy sleep |  |  |  |  |  |  | 0.302 |
| Unhealthy | 2,191/16,287 | 1.00 [Reference] | 1.03 (0.93-1.13) | 0.610 | 0.90 (0.81-1.00) | 0.049 |  |
| Healthy | 1,264/14,761 | 1.00 [Reference] | 0.85 (0.75-0.97) | 0.018 | 0.80 (0.7-0.91) | 0.001 |  |

The analysis was performed in Model 2 (adjusted with for age, sex, ethnicity, household income, education, employed, smoking, alcohol consumption, physical activity (MET), healthy diet pattern, and healthy sleep pattern).

**Abbreviations:** MET, metabolic equivalent of task.

**Table S12.** Associations between nature exposures at 300 or 1000 m buffer and incidence of mental disorders among people with type 2 diabetes (N = 37,519).

|  | **300 m buffer** | | | | **1000 m buffer** | | | |
| --- | --- | --- | --- | --- | --- | --- | --- | --- |
|  | **N** | **Cases/Person-years** | **HR (95% CI)** | **P-Value** | **N** | **Cases/Person-years** | **HR (95% CI)** | **P-Value** |
| **Green space** |  |  |  |  |  |  |  |  |
| Tertile 1 | 12,473 | 1,480/93,910 | Reference |  | 12,450 | 1,599/93,834 | Reference |  |
| Tertile 2 | 12,503 | 1,523/94,674 | 1.00 (0.93-1.08) | 0.965 | 12,548 | 1,469/95,051 | 0.90 (0.84-0.97) | 0.006 |
| Tertile 3 | 12,543 | 1,379/95,260 | 0.93 (0.87-1.01) | 0.075 | 12,521 | 1,314/94,959 | 0.83 (0.77-0.90) | 0.000 |
| P for trend |  |  |  | 0.077 |  |  |  | <0.001 |
| **Natural environment** |  |  |  |  |  |  |  |  |
| Tertile 1 | 12,610 | 1,587/94,672 | Reference |  | 12,470 | 1,616/94,009 | Reference |  |
| Tertile 2 | 12,454 | 1,447/93,963 | 0.93 (0.86-1.00) | 0.037 | 12,553 | 1,472/95,021 | 0.91 (0.84-0.97) | 0.007 |
| Tertile 3 | 12,455 | 1,348/95,210 | 0.87 (0.81-0.94) | <0.001 | 12,496 | 1,294/94,814 | 0.82 (0.76-0.89) | <0.001 |
| P for trend |  |  |  | <0.001 |  |  |  | <0.001 |
| **Garden space** |  |  |  |  |  |  |  |  |
| Tertile 1 | 12,489 | 1,553/93,831 | Reference |  | 12,490 | 1,519/94,418 | Reference |  |
| Tertile 2 | 12,549 | 1,517/95,822 | 0.97 (0.90-1.04) | 0.385 | 12,548 | 1,449/94,996 | 0.96 (0.89-1.03) | 0.229 |
| Tertile 3 | 12,481 | 1,312/94,191 | 0.87 (0.81-0.94) | <0.001 | 12,481 | 1,414/94,430 | 0.95 (0.89-1.03) | 0.214 |
| P for trend |  |  |  | <0.001 |  |  |  | 0.210 |

The analysis was performed in Model 2 (adjusted with for age, sex, ethnicity, household income, education, employed, smoking, alcohol consumption, physical activity (MET), healthy diet pattern, and healthy sleep pattern).

**Abbreviations:** CI, confidence interval; HR, hazard ratio; MET, metabolic equivalent of task.

**Table S13.** Associations between nature exposures at 300 or 1000 m buffer and incidence of mental disorders among people without diabetes (N = 410,338).

|  | **300 m buffer** | | | | **1000 m buffer** | | | |
| --- | --- | --- | --- | --- | --- | --- | --- | --- |
|  | **N** | **Cases/Person-years** | **HR (95% CI)** | **P-Value** | **N** | **Cases/Person-years** | **HR (95% CI)** | **P-Value** |
| **Green space** |  |  |  |  |  |  |  |  |
| Tertile 1 | 136,790 | 12,900/1,759,201 | Reference |  | 136,796 | 13,311/1,751,886 | Reference |  |
| Tertile 2 | 136,769 | 13,705/1,771,627 | 1.01 (0.98-1.03) | 0.610 | 136,766 | 13,368/1,777,232 | 0.94 (0.92-0.97) | <0.001 |
| Tertile 3 | 136,779 | 11,997/1,788,207 | 0.91 (0.89-0.94) | <0.001 | 136,776 | 11,923/1,789,917 | 0.87 (0.85-0.90) | <0.001 |
| P for trend |  |  |  | 0.006 |  |  |  | <0.001 |
| **Natural environment** |  |  |  |  |  |  |  |  |
| Tertile 1 | 136,920 | 13,422/1,757,084 | Reference |  | 136,875 | 13,665/1,753,271 | Reference |  |
| Tertile 2 | 137,478 | 13,296/1,785,450 | 0.95 (0.93-0.97) | <0.001 | 136,711 | 13,205/1,777,249 | 0.92 (0.90-0.95) | <0.001 |
| Tertile 3 | 135,940 | 11,884/1,776,501 | 0.88 (0.86-0.91) | <0.001 | 136,752 | 11,732/1,788,515 | 0.85 (0.83-0.87) | <0.001 |
| P for trend |  |  |  | 0.006 |  |  |  | <0.001 |
| **Garden space** |  |  |  |  |  |  |  |  |
| Tertile 1 | 136,789 | 13,032/1,778,909 | Reference |  | 136,787 | 12,773/1,785,209 | Reference |  |
| Tertile 2 | 136,774 | 13,338/1,771,225 | 1.02 (1.00-1.05) | 0.089 | 136,778 | 13,378/1,775,228 | 1.04 (1.01-1.06) | 0.003 |
| Tertile 3 | 136,775 | 12,232/1,768,901 | 0.96 (0.94-0.98) | 0.001 | 136,773 | 12,451/1,758,598 | 1.01 (0.99-1.04) | 0.354 |
| P for trend |  |  |  | 0.002 |  |  |  | 0.338 |

The analysis was performed in Model 2 (adjusted with for age, sex, ethnicity, household income, education, employed, smoking, alcohol consumption, physical activity (MET), healthy diet pattern, and healthy sleep pattern).

**Abbreviations:** CI, confidence interval; HR, hazard ratio; MET, metabolic equivalent of task.

**Table S14.** Associations between nature exposures at 300 or 1000 m buffer and incidence of mental disorders among people with diabetes who had lived at the current address more than 10 years before baseline (N = 27,327).

|  | **300 m buffer** | | | | **1000 m buffer** | | | |
| --- | --- | --- | --- | --- | --- | --- | --- | --- |
|  | **N** | **Cases/Person-years** | **HR (95% CI)** | **P-Value** | **N** | **Cases/Person-years** | **HR (95% CI)** | **P-Value** |
| **Green space** |  |  |  |  |  |  |  |  |
| Tertile 1 | 9,166 | 957/68,912 | Reference |  | 8,837 | 996/66,481 | Reference |  |
| Tertile 2 | 9,000 | 963/67,831 | 1.01 (0.93-1.11) | 0.793 | 9,225 | 967/69,368 | 0.93 (0.85-1.01) | 0.093 |
| Tertile 3 | 9,161 | 916/69,439 | 0.96 (0.88-1.05) | 0.389 | 9,265 | 873/70,332 | 0.84 (0.77-0.93) | <0.001 |
| P for trend |  |  |  | 0.391 |  |  |  | <0.001 |
| **Natural environment** |  |  |  |  |  |  |  |  |
| Tertile 1 | 9,076 | 993/67,859 | Reference |  | 8,777 | 997/66,023 | Reference |  |
| Tertile 2 | 9,089 | 945/68,546 | 0.95 (0.87-1.04) | 0.293 | 9,298 | 978/70,103 | 0.93 (0.85-1.02) | 0.108 |
| Tertile 3 | 9,162 | 898/69,776 | 0.90 (0.82-0.99) | 0.025 | 9,252 | 861/70,055 | 0.84 (0.76-0.92) | <0.001 |
| P for trend |  |  |  | 0.025 |  |  |  | <0.001 |
| **Garden space** |  |  |  |  |  |  |  |  |
| Tertile 1 | 8,490 | 920/63,632 | Reference |  | 8,725 | 933/65,843 | Reference |  |
| Tertile 2 | 9,132 | 995/69,349 | 1.01 (0.92-1.11) | 0.814 | 9,188 | 944/69,473 | 0.97 (0.88-1.06) | 0.449 |
| Tertile 3 | 9,705 | 921/73,200 | 0.90 (0.82-0.99) | 0.023 | 9,414 | 959/70,865 | 0.97 (0.89-1.07) | 0.555 |
| P for trend |  |  |  | 0.021 |  |  |  | 0.559 |

The analysis was performed in Model 2 (adjusted with for age, sex, ethnicity, household income, education, employed, smoking, alcohol consumption, physical activity (MET), healthy diet pattern, and healthy sleep pattern).

**Abbreviations:** CI, confidence interval; HR, hazard ratio; MET, metabolic equivalent of task.

**Table S15.** Associations between nature exposures at 300 or 1000 m buffer and incidence of mental disorders among people with diabetes after excluding participants with mental disorders events within 1 years of follow-up (N = 38,833).

|  | **300 m buffer** | | | | **1000 m buffer** | | | |
| --- | --- | --- | --- | --- | --- | --- | --- | --- |
|  | **N** | **Cases/Person-years** | **HR (95% CI)** | **P-Value** | **N** | **Cases/Person-years** | **HR (95% CI)** | **P-Value** |
| **Green space** |  |  |  |  |  |  |  |  |
| Tertile 1 | 12,935 | 1,323/98,511 | Reference |  | 12,927 | 1,443/98,579 | Reference |  |
| Tertile 2 | 12,956 | 1,390/99,070 | 1.03 (0.95-1.11) | 0.517 | 12,957 | 1,328/99,070 | 0.91 (0.84-0.98) | 0.016 |
| Tertile 3 | 12,942 | 1,236/99,517 | 0.94 (0.87-1.02) | 0.123 | 12,949 | 1,178/99,450 | 0.83 (0.76-0.90) | <0.001 |
| P for trend | - | - | - | 0.128 | - | - | - | <0.001 |
| **Natural environment** |  |  |  |  |  |  |  |  |
| Tertile 1 | 13,063 | 1,432/99,269 | Reference |  | 12,932 | 1,456/98,620 | Reference |  |
| Tertile 2 | 12,880 | 1,311/98,179 | 0.93 (0.86-1.01) | 0.068 | 12,950 | 1,328/99,073 | 0.91 (0.84-0.98) | 0.015 |
| Tertile 3 | 12,890 | 1,206/99,651 | 0.86 (0.80-0.93) | <0.001 | 12,951 | 1,165/99,405 | 0.82 (0.76-0.89) | <0.001 |
| P for trend | - | - | - | <0.001 | - | - | - | <0.001 |
| **Garden space** |  |  |  |  |  |  |  |  |
| Tertile 1 | 12,918 | 1,385/98,446 | Reference |  | 12,923 | 1,356/99,067 | Reference |  |
| Tertile 2 | 12,943 | 1,372/99,813 | 0.99 (0.92-1.07) | 0.774 | 12,954 | 1,313/98,974 | 0.98 (0.91-1.05) | 0.563 |
| Tertile 3 | 12,972 | 1,192/98,839 | 0.89 (0.82-0.96) | 0.003 | 12,956 | 1,280/99,058 | 0.97 (0.90-1.05) | 0.421 |
| P for trend | - | - | - | 0.003 | - | - | - | 0.419 |

The analysis was performed in Model 2 (adjusted with for age, sex, ethnicity, household income, education, employed, smoking, alcohol consumption, physical activity (MET), healthy diet pattern, and healthy sleep pattern).

**Abbreviations:** CI, confidence interval; HR, hazard ratio; MET, metabolic equivalent of task.

**Table S16.** Associations between nature exposures at 300 or 1000 m buffer and incidence of mental disorders among people with diabetes after excluding the participants with missing data on covariates (N = 19,679).

|  | **300 m buffer** | | | | **1000 m buffer** | | | |
| --- | --- | --- | --- | --- | --- | --- | --- | --- |
|  | **N** | **Cases/Person-years** | **HR (95% CI)** | **P-Value** | **N** | **Cases/Person-years** | **HR (95% CI)** | **P-Value** |
| **Green space** |  |  |  |  |  |  |  |  |
| Tertile 1 | 6,560 | 717/49,423 | Reference |  | 6,560 | 779/49,130 | Reference |  |
| Tertile 2 | 6,559 | 711/49,109 | 0.98 (0.88-1.09) | 0.719 | 6,559 | 680/49,490 | 0.87 (0.78-0.96) | 0.007 |
| Tertile 3 | 6,560 | 666/50,004 | 0.94 (0.85-1.05) | 0.264 | 6,560 | 635/49,915 | 0.83 (0.74-0.92) | <0.001 |
| P for trend | - | - | - | 0.265 | - | - | - | <0.001 |
| **Natural environment** |  |  |  |  |  |  |  |  |
| Tertile 1 | 6,593 | 766/49,125 | Reference |  | 6,567 | 749/49,167 | Reference |  |
| Tertile 2 | 6,540 | 681/49,331 | 0.89 (0.80-0.99) | 0.026 | 6,553 | 730/49,483 | 0.97 (0.88-1.08) | 0.615 |
| Tertile 3 | 6,546 | 647/50,079 | 0.86 (0.77-0.95) | 0.004 | 6,559 | 615/49,886 | 0.84 (0.75-0.94) | 0.002 |
| P for trend | - | - | - | 0.004 | - | - | - | 0.002 |
| **Garden space** |  |  |  |  |  |  |  |  |
| Tertile 1 | 6,560 | 747/49,440 | Reference |  | 6,560 | 728/49,373 | Reference |  |
| Tertile 2 | 6,560 | 736/49,753 | 0.99 (0.90-1.10) | 0.893 | 6,562 | 680/49,819 | 0.93 (0.84-1.04) | 0.191 |
| Tertile 3 | 6,559 | 611/49,343 | 0.85 (0.76-0.94) | 0.002 | 6,557 | 686/49,343 | 0.97 (0.87-1.07) | 0.526 |
| P for trend | - | - | - | 0.003 | - | - | - | 0.516 |

The analysis was performed in Model 2 (adjusted with for age, sex, ethnicity, household income, education, employed, smoking, alcohol consumption, physical activity (MET), healthy diet pattern, and healthy sleep pattern).

**Abbreviations:** CI, confidence interval; HR, hazard ratio; MET, metabolic equivalent of task.

**Table S17.** Crude hazard ratios and 95 % confidence interval of incident mental disorders associated with nature exposures at 300 or 1000 m buffer among people with diabetes (N = 39,397).

|  | **300 m buffer** | | | | **1000 m buffer** | | | |
| --- | --- | --- | --- | --- | --- | --- | --- | --- |
|  | **N** | **Cases/Person-years** | **HR (95% CI)** | **P-Value** | **N** | **Cases/Person-years** | **HR (95% CI)** | **P-Value** |
| **Green space** |  |  |  |  |  |  |  |  |
| Tertile 1 | 13,133 | 1,521/98,595 | Reference |  | 13,133 | 1,649/98,669 | Reference |  |
| Tertile 2 | 13,132 | 1,566/99,156 | 1.02 (0.95-1.10) | 0.555 | 13,132 | 1,503/99,157 | 0.91 (0.84-0.97) | 0.005 |
| Tertile 3 | 13,132 | 1,426/99,592 | 0.93 (0.86-1.00) | 0.036 | 13,132 | 1,361/99,516 | 0.82 (0.76-0.88) | <0.001 |
| P for trend | - | - | - | 0.038 | - | - | - | <0.001 |
| **Natural environment** |  |  |  |  |  |  |  |  |
| Tertile 1 | 13,263 | 1,632/99,356 | Reference |  | 13,138 | 1,662/98,714 | Reference |  |
| Tertile 2 | 13,051 | 1,482/98,257 | 0.92 (0.85-0.98) | 0.015 | 13,128 | 1,506/99,154 | 0.90 (0.84-0.97) | 0.003 |
| Tertile 3 | 13,083 | 1,399/99,729 | 0.85 (0.79-0.91) | <0.001 | 13,131 | 1,345/99,474 | 0.80 (0.75-0.86) | <0.001 |
| P for trend | - | - | - | <0.001 | - | - | - | <0.001 |
| **Garden space** |  |  |  |  |  |  |  |  |
| Tertile 1 | 13,133 | 1,600/98,540 | Reference |  | 13,134 | 1,567/99,151 | Reference |  |
| Tertile 2 | 13,133 | 1,562/99,899 | 0.96 (0.90-1.03) | 0.277 | 13,132 | 1,491/99,062 | 0.95 (0.89-1.02) | 0.189 |
| Tertile 3 | 13,131 | 1,351/98,904 | 0.84 (0.78-0.90) | <0.001 | 13,131 | 1,455/99,130 | 0.93 (0.87-1.00) | 0.047 |
| P for trend | - | - | - | <0.001 | - | - | - | 0.047 |

The analysis was performed based on unadjusted model.

**Abbreviations**: CI, confidence interval; HR, hazard ratio.

**Table S18.** Associations between nature exposures at 300 m buffer and incidence of mental disorders among people with diabetes with adjustment air pollutants (N = 38,983).

|  | Adjusted by NO_2_ | | Adjusted by NO_x_ | | Adjusted by PM_10_ | | Adjusted by PM_2.5_ | | Adjusted by All | |
| --- | --- | --- | --- | --- | --- | --- | --- | --- | --- | --- |
|  | **HR (95% CI)** | **P-Value** | **HR (95% CI)** | **P-Value** | **HR (95% CI)** | **P-Value** | **HR (95% CI)** | **P-Value** | **HR (95% CI)** | **P-Value** |
| **Green space** |  |  |  |  |  |  |  |  |  |  |
| Tertile 1 | Reference |  | Reference |  | Reference |  | Reference |  | Reference |  |
| Tertile 2 | 1.04 (0.97-1.12) | 0.240 | 1.03 (0.96-1.11) | 0.404 | 1.01 (0.94-1.09) | 0.683 | 1.04 (0.97-1.12) | 0.260 | 1.05 (0.98-1.13) | 0.184 |
| Tertile 3 | 1.04 (0.96-1.13) | 0.362 | 1.00 (0.93-1.09) | 0.943 | 0.96 (0.89-1.04) | 0.333 | 1.05 (0.97-1.14) | 0.258 | 1.07 (0.98-1.17) | 0.113 |
| P for trend | - | 0.336 | - | 0.912 | - | 0.349 | - | 0.239 | - | 0.103 |
| **Natural environment** | |  |  |  |  |  |  |  |  |  |
| Tertile 1 | Reference |  | Reference |  | Reference |  | Reference |  | Reference |  |
| Tertile 2 | 0.96 (0.89-1.03) | 0.289 | 0.95 (0.88-1.02) | 0.166 | 0.93 (0.87-1.00) | 0.059 | 0.96 (0.89-1.04) | 0.313 | 0.97 (0.90-1.04) | 0.378 |
| Tertile 3 | 0.95 (0.87-1.03) | 0.216 | 0.92 (0.85-1.00) | 0.055 | 0.89 (0.82-0.96) | 0.003 | 0.96 (0.88-1.05) | 0.375 | 0.97 (0.89-1.06) | 0.525 |
| P for trend | - | 0.208 | - | 0.053 | - | 0.003 | - | 0.353 | - | 0.499 |
| **Garden space** |  |  |  |  |  |  |  |  |  |  |
| Tertile 1 | Reference |  | Reference |  | Reference |  | Reference |  | Reference |  |
| Tertile 2 | 0.97 (0.90-1.04) | 0.398 | 0.98 (0.91-1.05) | 0.500 | 0.97 (0.91-1.04) | 0.415 | 0.97 (0.91-1.04) | 0.410 | 0.96 (0.90-1.03) | 0.314 |
| Tertile 3 | 0.86 (0.80-0.93) | <0.001 | 0.87 (0.81-0.94) | <0.001 | 0.86 (0.80-0.93) | <0.001 | 0.87 (0.81-0.93) | <0.001 | 0.86 (0.80-0.92) | <0.001 |
| P for trend | - | <0.001 | - | <0.001 | - | <0.001 | - | <0.001 | - | <0.001 |

The analysis was performed in Model 2 (adjusted with for age, sex, ethnicity, household income, education, employed, smoking, alcohol consumption, physical activity (MET), healthy diet pattern, and healthy sleep pattern).

**Abbreviations:** CI, confidence interval; HR, hazard ratio; MET, metabolic equivalent of task; NO_2_, nitrogen dioxide; NO_x_, nitrogen oxides; PM_10_, particulate matter (PM) with aerodynamic diameter ≤10 µm; PM_2.5_, PM with aerodynamic diameter < 2.5 µm.

**Table S19.** Associations between nature exposures at 1000 m buffer and incidence of mental disorders among people with diabetes with adjustment air pollutants (N = 38,983).

|  | Adjusted by NO_2_ | | Adjusted by NO_x_ | | Adjusted by PM_10_ | | Adjusted by PM_2.5_ | | Adjusted by All | |
| --- | --- | --- | --- | --- | --- | --- | --- | --- | --- | --- |
|  | **HR (95% CI)** | **P-Value** | **HR (95% CI)** | **P-Value** | **HR (95% CI)** | **P-Value** | **HR (95% CI)** | **P-Value** | **HR (95% CI)** | **P-Value** |
| **Green space** |  |  |  |  |  |  |  |  |  |  |
| Tertile 1 | Reference |  | Reference |  | Reference |  | Reference |  | Reference |  |
| Tertile 2 | 0.93 (0.87-1.01) | 0.075 | 0.92 (0.86-0.99) | 0.031 | 0.91 (0.85-0.98) | 0.010 | 0.93 (0.87-1.01) | 0.068 | 0.93 (0.86-1.01) | 0.085 |
| Tertile 3 | 0.89 (0.81-0.98) | 0.019 | 0.87 (0.80-0.95) | 0.001 | 0.85 (0.78-0.91) | 0.001 | 0.90 (0.83-0.98) | 0.020 | 0.90 (0.81-1.00) | 0.056 |
| P for trend | - | 0.017 | - | 0.001 | - | <0.001 | - | 0.018 | - | 0.050 |
| **Natural environment** | |  |  |  |  |  |  |  |  |  |
| Tertile 1 | Reference |  | Reference |  | Reference |  | Reference |  | Reference |  |
| Tertile 2 | 0.93 (0.86-1.01) | 0.076 | 0.92 (0.86-0.99) | 0.033 | 0.91 (0.85-0.98) | 0.01 | 0.94 (0.87-1.01) | 0.076 | 0.93 (0.86-1.01) | 0.085 |
| Tertile 3 | 0.88 (0.80-0.97) | 0.011 | 0.86 (0.79-0.94) | 0.001 | 0.84 (0.78-0.91) | <0.001 | 0.89 (0.82-0.98) | 0.014 | 0.89 (0.80-0.99) | 0.038 |
| P for trend | - | 0.010 | - | <0.001 | - | <0.001 | - | 0.013 | - | 0.035 |
| **Garden space** |  |  |  |  |  |  |  |  |  |  |
| Tertile 1 | Reference |  | Reference |  | Reference |  | Reference |  | Reference |  |
| Tertile 2 | 0.93 (0.87-1.00) | 0.062 | 0.95 (0.88-1.02) | 0.147 | 0.95 (0.89-1.02) | 0.198 | 0.94 (0.87-1.01) | 0.073 | 0.92 (0.86-0.99) | 0.029 |
| Tertile 3 | 0.92 (0.85-0.99) | 0.019 | 0.94 (0.88-1.01) | 0.096 | 0.94 (0.88-1.02) | 0.121 | 0.93 (0.87-1.00) | 0.053 | 0.90 (0.84-0.98) | 0.009 |
| P for trend | - | 0.019 | - | 0.095 | - | 0.120 | - | 0.053 | - | 0.009 |

The analysis was performed in Model 2 (adjusted with for age, sex, ethnicity, household income, education, employed, smoking, alcohol consumption, physical activity (MET), healthy diet pattern, and healthy sleep pattern).

**Abbreviations:** CI, confidence interval; HR, hazard ratio; MET, metabolic equivalent of task; NO_2_, nitrogen dioxide; NO_x_, nitrogen oxides; PM_10_, particulate matter (PM) with aerodynamic diameter ≤10 µm; PM_2.5_, PM with aerodynamic diameter < 2.5 µm.
